# Exploring the structural lexicon of the Proteome via Metric Geometry

**DOI:** 10.1371/journal.pcbi.1014487

**Published:** 2026-06-30

**Authors:** Elijah Gunther, Pablo G. Camara

**Affiliations:** 1 Department of Genetics, Perelman School of Medicine, University of Pennsylvania, Philadelphia, Pennsylvania, United States of America; 2 Institute for Biomedical Informatics, Perelman School of Medicine, University of Pennsylvania, Philadelphia, Pennsylvania, United States of America; 3 AI2D Center for Artificial Intelligence and Data Science for Integrated Diagnostics, Perelman School of Medicine, University of Pennsylvania, Philadelphia, Pennsylvania, United States of America; Penn State University: The Pennsylvania State University, UNITED STATES OF AMERICA

## Abstract

The three-dimensional structure of proteins is intimately linked to their function, yet establishing comprehensive frameworks for systematically comparing and organizing protein structures across the proteome remains a significant challenge. Here, we introduce GWProt, a computational framework that leverages recent advances in metric geometry, such as Gromov-Wasserstein couplings, for protein structure alignment and analysis. GWProt enables the integration of biochemical information into structural comparisons and introduces the concept of local geometric distortion, a measure that captures local conformational differences. We demonstrate the utility of this framework by identifying conformational switches within individual proteins, detecting functional domains shared among evolutionarily distant viral proteins, revealing topological rearrangements in homologous folds, and uncovering recurrent short structural motifs underlying functional domains across the human proteome. Collectively, these results establish the use of metric geometry as a versatile and quantitative framework for the systematic comparative analysis of protein structures, complementing existing approaches for elucidating protein organization.

## Author summary

Proteins carry out their functions through their three-dimensional shapes, but systematically comparing those shapes across many proteins remains challenging. In this study, we introduce GWProt, an open-source computational framework that applies ideas from metric geometry to protein structure analysis. GWProt aligns proteins in a flexible, quantitative way that does not depend on the order of amino acids in the sequence, and it can measure both overall structural similarity and local differences within a protein. Using this approach, we show that GWProt can identify flexible switch regions in the cancer-related protein KRAS, detect conserved catalytic regions in highly diverse viral enzymes, and recognize structural rearrangements that are difficult for many standard methods to capture. When we applied GWProt to thousands of human protein domains, we found a shared “structural lexicon” of short recurring 3D motifs associated with protein stability, catalytic function, and disease-causing mutations. Together, these results show that metric geometry provides a powerful framework for organizing and interpreting protein structure space.

## Introduction

Proteins are the fundamental information-processing units of all living systems, with their functional capabilities determined by the dynamic 3D conformations encoded in their amino acid sequences. Understanding the diversity and governing principles of protein structure is therefore of central importance, and substantial efforts have been devoted over the past four decades to developing algorithms for protein structure alignment [1], yielding widely adopted methods such as DALI [2], SSAP [3], CE [4], and, more recently, TM-align [5], Foldseek [6], and Reseek [7]. The relevance of these approaches has grown in parallel with the rapid expansion of the Protein Data Bank [8] (PDB), which now houses hundreds of thousands of experimentally determined protein structures, and has been further amplified by recent advances in deep learning that enable accurate prediction of protein structures directly from sequence [9–11]. These developments have highlighted the modular design of proteins and facilitated the identification and systematic classification of functional protein domains [12–16]. Additionally, they have led to the recognition of smaller recurrent building blocks of protein architecture, such as super-secondary structural elements [17–21] and 3D microenvironments [22–24], although their systematic classification remains incomplete, partially due to the lack of a unifying organizational framework.

From a mathematical standpoint, the study and comparison of geometric structures fall within the discipline of metric geometry [25]. The primary objects of study in metric geometry are metric spaces: collections of points equipped with a notion of distance between them. Since the introduction of the modern concept of metric space by Maurice Fréchet in the early twentieth century [26], the field has undergone several major developments. In 1981, Mikhail Gromov transformed it with the introduction of the Gromov-Hausdorff distance [27], which provided a rigorous framework for quantitatively comparing metric spaces independently of rigid transformations such as rotations and translations. However, because computing the Gromov-Hausdorff distance involves solving an optimization problem of exponential complexity in the number of points, its impact initially remained largely theoretical, with few practical applications. Over the past decade, this has changed with the development of rigorous and computationally efficient relaxations, such as the Gromov-Wasserstein distance [28–30], which reformulates the problem within the framework of optimal transport theory and can be approximated in nearly linear time [31–33]. These advances have established a mathematical foundation for the quantitative study and comparison of geometric structures encountered in real-world applications, including cell morphometry [34], single-cell and spatial omics [35–39], neuroscience [40], and communication networks [41].

In this work, we investigate the application of these advances in metric geometry to the problem of protein structure alignment and extend them by introducing the notion of local geometric distortion. Our results demonstrate that metric geometry provides a powerful and versatile framework for the systematic comparative analysis of protein structures, complementing existing approaches with several strengths, such as the ability to detect conserved structural motifs, the capacity to incorporate biochemical information into structural comparisons, and robustness to topologically non-trivial sequence rearrangements. We demonstrate this framework with the analysis of evolutionarily distant viral proteins, and apply it to the human proteome, where we show that metric geometry can serve as an organizing principle for the systematic study of recurrent structural polypeptide fragments.

To facilitate use, we have implemented and documented the computational methods of this work as an open-source software package, available to the broader community. Together, our results establish the use of metric geometry as a complementary quantitative framework for advancing the understanding of protein organization, function, and evolution.

## Results

### GWProt: Gromov-Wasserstein correspondences for protein structural alignment

**Fig 1:**
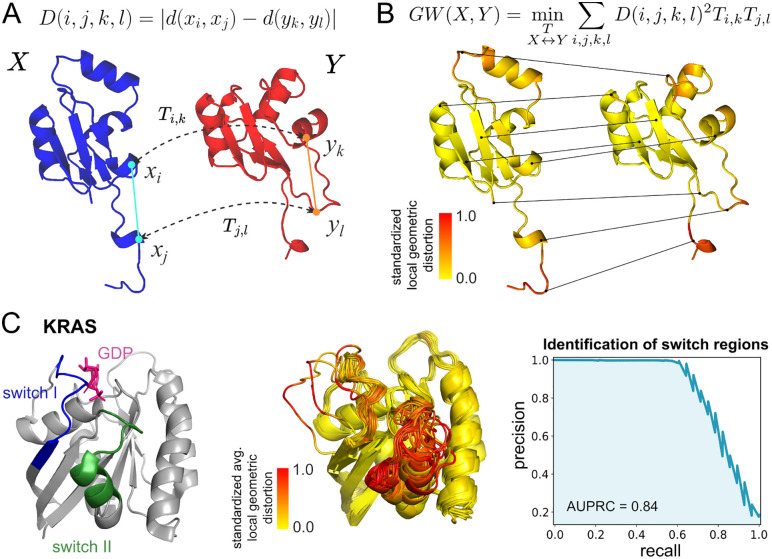
Gromov-Wasserstein correspondences for protein structural alignment. **A)** Schematic illustrating the concept of geometric distortion underlying Gromov-Hausdorff and GW distances. Given a correspondence T between two protein structures X and Y, the geometric distortion D(i,j,k,l) between a pair of C_α_ atoms $x_{i}$ and $x_{j}$ in protein X and their matched α-carbons $y_{k}$ and $y_{l}$ in protein Y is defined as the discrepancy between the Euclidean distances $d(x_{i},x_{j})$ and $d(y_{k},y_{l})$. **B)** The GW correspondence between two protein structures is the mapping T that minimizes the total geometric distortion. The local geometric distortion quantifies the contribution of each C_α_ atom to the optimal cost GW(X,Y). In the figure, the optimal GW correspondence is visualized for two proteins, with colors indicating local geometric distortion, showing regions of high (red) and low (yellow) structural agreement. **C)** Identification of switch regions in KRAS. Left: Backbone of the KRAS protein with annotated switch I and switch II regions. Middle: GW-based alignment of 54 experimentally determined KRAS structures, colored by average local geometric distortion. The switch regions show high structural variability. Right: Median precision-recall curve for a predictor of switch regions based on the average local geometric distortion. AUPRC: area under precision-recall curve.

The problem of establishing a local correspondence between two shapes, or *metric spaces*, is a central problem in metric geometry. The Gromov-Hausdorff distance between two metric spaces establishes an optimal correspondence between their points by minimizing a loss function that quantifies the total geometric distortion induced by the mapping [28,30] (Fig 1A). The value of the loss function at its minimum defines a structural distance, satisfying the triangle inequality and all other mathematical properties required of a distance function. Intuitively, the Gromov-Hausdorff distance captures the minimal deformation needed to transform one shape into another. Thus, it provides a natural and rigorous mathematical framework for structurally aligning proteins, allowing us to determine optimal residue-to-residue pairings between two protein structures (Fig 1B).

The computation of Gromov-Hausdorff correspondences is an NP-hard problem, making it computationally unfeasible for most practical applications. However, efficient relaxations can be achieved by using probabilistic correspondences, as they enable reformulating the problem in terms of optimal transport theory [28–30]. The Gromov-Wasserstein (GW) distance approximates the Gromov-Hausdorff distance and provides a measure of structural similarity by identifying an optimal weighted correspondence between the two shapes (Fig 1B, Methods). Like the Gromov-Hausdorff distance, the GW distance is a true distance function.

The computation of GW correspondences (more commonly referred to in the mathematical literature as GW couplings) requires solving a non-convex optimization problem that can be computationally demanding. When the two shapes are of comparable size, exact solvers are often described as having roughly cubic time complexity in the number of sampled points. In practice, however, efficient algorithms often perform substantially better than such asymptotic estimates, and several recent approximations achieve near-linear runtime in specific settings [32,42–44]. Recent extensions, such as fused and unbalanced variants [40,45,46], also allow additional features in the correspondences (e.g., local biochemical properties) to be incorporated and support partial structural alignments.

To investigate the utility of GW correspondences for protein structure alignment, we developed a Python package, GWProt, which computes GW and fused GW couplings between the sets of C_α_ atoms of protein pairs. Additionally, by expressing the GW distance as a sum over individual residue contributions, we introduced the concept of local geometric distortion, a measure of the degree of structural conservation for each residue in the alignment (Fig 1B, Methods). Unlike the residue-level deviations underlying root-mean-squared deviation (RMSD), local geometric distortion is defined in terms of distances between residues within each protein and is well defined for soft (probabilistic) alignments. GWProt is freely available to the community as open-source software (see Code Availability).

### Local geometric distortion identifies active sites in the KRAS oncoprotein

As an initial proof of concept to evaluate the utility of GW correspondences for structural protein alignment, we analyzed 54 crystallographic structures of the human GTPase KRAS (Kristen Rat Sarcoma), including both wild-type and mutant forms, determined by X-ray diffraction and obtained from the RCSB Protein Data Bank [8] (S1 Table).

KRAS cycles between an inactive guanosine diphosphate (GDP)-bound and an active guanosine triphosphate (GTP)-bound state. In its GTP-bound form, it binds and activates various effector proteins within the RAS signaling pathway. The interface for effector binding is formed by two flexible regions, known as switch I and switch II [47] (Fig 1C).

We used GWProt to compute GW correspondences between all KRAS structures (including both GDP- and GTP-analogue-bound forms), as well as the local geometric distortion at each C_α_ atom (Fig 1C). As expected, the resulting correspondences were close to the identity map (Pearson’s correlation with the identity map, r=0.985±0.021), reflecting the high degree of homology among the crystallographic structures. Moreover, most of the local geometric distortion was concentrated in the two switch regions. Thus, using average distortion values alone, we were able to predict the location of the switch sites in each structure with an area under the precision-recall curve (AUPRC) of 0.84±0.03 (Fig 1C).

These results demonstrate that GW correspondences can efficiently capture local structural variation and identify conserved and variable regions across closely related protein conformations.

### Local geometric distortion identifies functional domains in evolutionarily distant viral proteins

**Fig 2:**
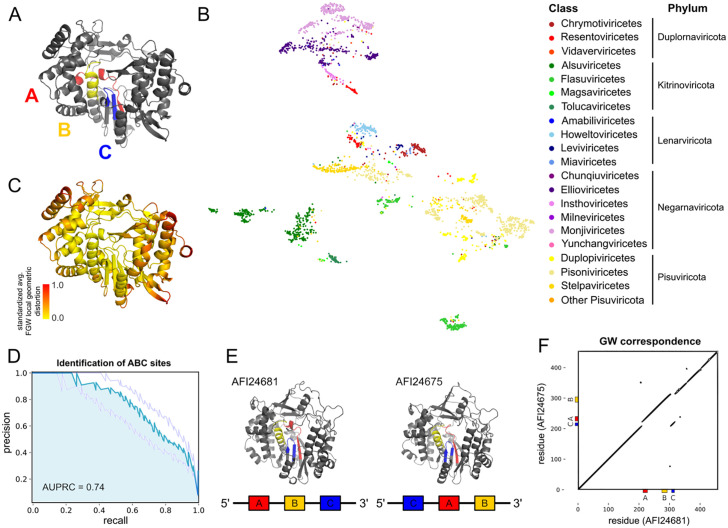
Identification of structurally conserved catalytic sites in RdRps. **A)** Hepacivirus hominis (GenPept ID AFD18577) RdRp core domain with A, B, and C motif sites highlighted. **B)** UMAP representation of the GW-based structural space of 2,777 RdRps. The UMAP is labeled by Ribovirus class, showing consistency between structural organization and viral taxonomy. **C)** Example of an RdRp core domain colored by average FGW local geometric distortion. Regions of low distortion, mostly located at the catalytic core, are structurally conserved. **D)** Median, 20%, and 80% percentile precision-recall curves for predicting A, B, or C sites based on average FGW local geometric distortion across 97 randomly selected RdRps. AUPRC: area under the precision-recall curve. **E)** Example of ABC and CAB RdRp core domains showing a high degree of structural homology. **F)** Dot plot visualization of the GW correspondence between the two proteins shown in **(E)**. The correspondence clearly captures the genomic circular permutation between the A, B, and C motifs.

Having assessed the utility of GW correspondences for identifying structurally variable regions among closely related protein conformations, we next turned our attention to identifying structurally conserved regions among distantly related proteins. For that purpose, we analyzed the computationally predicted structures of the core domain of 2,777 RNA-dependent RNA-polymerases (RdRps) from Riboviruses spanning 21 taxonomic classes [48,49] (S2 Table). RdRp serves as the principal marker for the higher-rank classification of RNA viruses that lack a DNA stage of replication [48–50]. Its core domain binds the RNA strand and comprises palm, fingers, and thumb subdomains [51], arranged in a fashion analogous to a hand gripping the strand. The palm subdomain contains the catalytic motifs A, B, and C, which are highly conserved among most known RdRps [52] (Fig 2A). However, although the palm subdomain is structurally conserved, its amino acid sequence identity can be as low as 10% [53].

We aligned the RdRp structures using GW correspondences and visualized the resulting structural distances using Uniform Manifold Approximation and Projection [54] (UMAP) (Fig 2B). In this representation, RdRps from different viral classes clustered distinctly, indicating that RdRp structural space recapitulates much of the current RNA virus taxonomy, which is derived primarily from RdRp evolutionary relationships [48–50]. To quantify the consistency of the GW structural space with the established class-level taxonomy, we trained a *k* = 3 nearest-neighbor classifier to predict viral class based on the GW distances to RdRp structures in the training set, achieving a Matthews correlation coefficient (MCC) of 0.94 (10-fold cross-validation).

We used the average local geometric distortion to identify structurally conserved regions in 97 randomly selected RdRps (Methods). The inner region, corresponding to the catalytic core, exhibited substantially lower distortion than the outer regions (S1A Fig), consistent with greater structural conservation. Local geometric distortion remained largely stable across different initializations of the GW optimization (S2 Fig) and was only weakly correlated with residue-level deviations from RMSD-minimizing rigid-body alignment (mean Spearman correlation ρ=0.15; S1B Fig), indicating that the two measures capture qualitatively different information. For each protein, we then computed a precision-recall curve to assess the accuracy of predicting the A, B, and C motif sites based on regions of low local geometric distortion (S1C Fig), using the output of Palmscan [48], a sequence-based software specifically designed to identify A, B, and C motifs, as the ground truth. The average AUPRC from this analysis was 0.72±0.11, indicating a strong degree of agreement between A, B, and C sites and low-distortion regions, and substantially exceeding the value obtained when residue-level deviations were used in place of local geometric distortion (AUPRC =0.44). This estimate is likely conservative, however, because other structurally conserved regions, such as D and E motifs, are not detected by Palmscan.

We then reasoned that incorporating biochemical information into the computation of GW correspondences and local geometric distortion could enhance the identification of functional sites. To test this, we recomputed the alignments using a fused GW approach incorporating the hydrophobicity of each residue in the computation of correspondences (Methods). This modification of our approach led to an improvement in the accuracy of A, B, and C site predictions, with an average AUPRC of 0.74±0.11 (Fig 2C and 2D; Wilcoxon signed rank test p-value = 3 x 10^-14^).

Collectively, these results demonstrate the utility of local geometric distortion as an unsupervised and interpretable method for identifying structurally conserved regions in evolutionarily distant proteins and show that this signal can be further enhanced by integrating orthogonal biochemical information into the computation of GW correspondences.

### GWProt structural alignment detects internal permutations in homologous proteins

Structural rearrangements facilitate the modular evolution of protein domains, with structural motifs often recurring in different combinations across proteins. Some of these rearrangements do not preserve the sequential order of motifs, with circular permutations of the entire protein being the most prominent example [55]. In certain cases, however, topologically non-trivial rearrangements affect only a small subset of the protein. For instance, in the RdRps of several ribovirus families, the conserved A, B, and C motifs appear in CAB order in the amino acid chain, rather than the canonical ABC sequence [56,57] (Fig 2E). Detecting such internal permutations poses a significant challenge, as most structural alignment methods rely on sequential ordering or are tailored to specific cases, such as circular permutations [58] or permuted RdRps [48]. Only a few general ordering-independent methods based on computer vision approaches like geometric hashing have been developed to address this problem [59–62].

Since GW correspondences minimize the geometric distortion between pairs of C_α_ atoms, they are independent of the sequence order, and the resulting structural alignments should be robust to internal permutations. To verify this, we examined the GW correspondences between the catalytic cores of ABC and CAB RdRps with high structural homology. As anticipated, the correspondences unequivocally identified the permutation of active sites and were consistent with maps produced by existing ordering-independent methods (Fig 2F and S3 Fig), confirming the robustness of GW correspondences to complex amino acid sequence rearrangements of structurally conserved motifs.

### GW distances support the discovery of previously unseen viral phyla

The value of the GW cost function at its minimum defines a distance function that can be used to quantify protein structure similarity [29]. Although numerous algorithms for protein structural similarity quantification have been developed and extensively optimized over the past decades [2–6], leaving little room for improvement in this area, for completeness, we also assessed the performance of GW distances in relation to state-of-the-art methods.

For that purpose, we considered the problem of identifying RdRp core domains from previously unseen ribovirus phyla. We supplemented our RdRp dataset with 300 computationally predicted structures from non-RdRp sequences (“decoys”), including eukaryotic, bacterial, retroelement-associated, and DNA-viral protein fragments, drawn from a curated structural benchmark assembled for the evaluation of viral palm-domain classifiers [48,49] (S2 Table). We then trained a nearest-neighbor classifier on a subset of RdRp core domain structures and decoys and tested its ability to distinguish decoys from RdRp core domain structures derived from individual phyla excluded from the training data (Methods). In this evaluation, the embedding space used by the classifier was constructed using either GWProt, Foldseek [6], TM-align [5], or rigid-body alignment minimizing RMSD [63]. A nearest-neighbor classifier is particularly well suited to this application because it depends only on the relative ordering of pairwise distances and is therefore relatively insensitive to normalization choices.

GWProt achieved the highest mean MCC across phyla, with a mean MCC of 0.953, compared with 0.927, 0.922, and 0.820 for TM-align, Foldseek, and RMSD, respectively, although it was not the top-performing method in any individual phylum (S4 Fig). Its higher overall average was driven primarily by more consistent performance across phyla, particularly in the identification of Lernaviricota and Negarnaviricota riboviria, where Foldseek and TM-align, respectively, showed reduced performance. These results indicate that GWProt performs comparably to current state-of-the-art methods while also providing complementary information for the identification of previously unseen viral phyla. Consistent with this interpretation, a majority-vote classifier combining GWProt, TM-align, and Foldseek achieved an average MCC of 0.981, outperforming any individual method (S4A Fig). These findings highlight the practical value of integrating complementary structural similarity measures to improve the identification of previously unseen phyla.

In these data, the runtime of GWProt scaled approximately as $n^{\text{2}.\text{3}}$, where n denotes the number of residues (S5 Fig). On a single thread of a standard desktop computer equipped with an 8-core 3.70 GHz Intel Xeon CPU, aligning a pair of proteins with 500 residues each took approximately 1 s. To assess whether downsampling could reduce runtime without substantially affecting accuracy, we repeated the analysis using 200, 100, or 50 regularly sampled residues from each RdRp core domain. MCC scores remained largely stable, with no noticeable loss of accuracy, whereas runtime for pairwise alignment across the full dataset decreased from about 2 days without downsampling to 25 min when 50 residues were used (S4B Fig). Results were also stable across different regular sampling offsets, although performance decreased noticeably when uniform random sampling was used instead of regular sampling (S4C Fig). Overall, these results show that downsampling can substantially reduce GWProt runtime in some analyses while preserving accuracy.

### Local geometric distortions across the human proteome reveal the structural lexicon underlying functional domains

Protein functional domains are composed of recurrent structural elements at different hierarchical levels. While the classification of functional domains is well established (e.g., via the CATH [12] and SCOP [13] databases), there is no comprehensive and systematic classification of the smaller structural motifs that make up functional domains beyond secondary structures, such as α-helices, β-sheets, and combinations of them. We therefore applied GWProt to systematically identify and characterize small structural motifs across protein domains catalogued in the CATH database.

**Fig 3:**
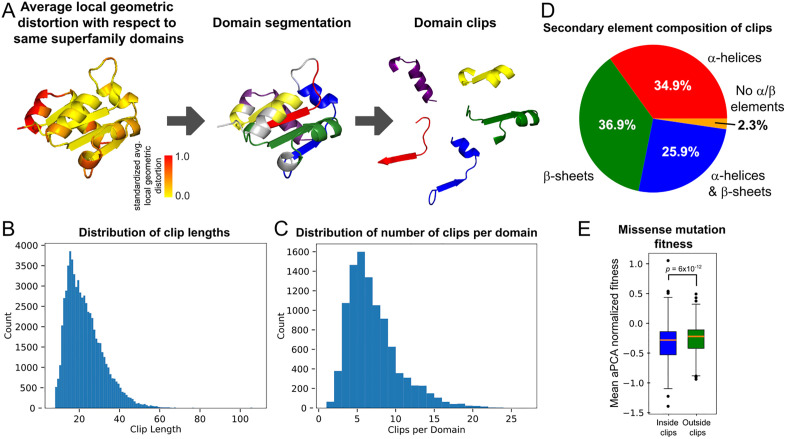
Structurally conserved fragments across human functional protein domains. **A)** Schematic of the approach. Each protein domain in the CATH database is aligned with all other domains belonging to the same homologous superfamily using GWProt, and the average local geometric distortion of each residue in the domain is computed. The average local geometric distortion is then used to segment structurally conserved domain clips. **B)** Distribution of domain clip lengths, measured in number of residues. **C)** Distribution of the number of clips per domain. **D)** Secondary structural element composition of domain clips. **E)** Box plot summarizing the distribution of mean normalized fitness of missense mutations inside domain clips and outside domain clips, but within the domain. aPCA: abundance protein fragment complementation assay.

For each CATH homologous superfamily with at least 5 elements (*n* = 552 superfamilies containing 10,139 domains), we computed pairwise GW correspondences and the local geometric distortions between the predicted structures of every element in the superfamily (Methods). We divided each domain into a set of short structural fragments or “clips” that are shared within its superfamily by removing regions with high average local geometric distortion (Fig 3A). This procedure yielded a total of 66,937 clips. The average length of each clip was 22.5 residues, and each domain contained, on average, 6.6 clips (Fig 3B and 3C, and S6 Fig). Most clips contained α-helices, β-sheets, or both, whereas only 2.3% lacked these elements (Fig 3D).

Like traditional local motifs [64–66], clips occupy a conceptual space between classical fixed-length backbone fragments [20,67,68] and full functional domains as classified by CATH [12] and SCOP [13], thereby bridging a gap in the structural hierarchy between secondary-structure elements and tertiary folds. However, unlike traditional local motifs, which are typically defined by geometric recurrence across structurally diverse proteins, clips are delineated through pairwise geometric comparison within homologous superfamilies, thereby capturing contiguous regions that have been structurally preserved across evolutionarily related domains.

Clips were significantly depleted of common (MAF ≥ 1%) missense single-nucleotide polymorphisms (SNPs) compared to other regions of the functional domain, suggesting that they are more likely to contain key active or structural sites (odds ratio = 0.93, Fisher’s exact test *p*-value = 0.004). Consistent with this, comparison with a site-saturation mutagenesis screen of 500 protein domains [69] revealed that missense mutations within the clips are more detrimental to protein stability than those outside the clips but still within the functional domain (Fig 3E; fold change in mean normalized fitness = 0.8, Wilcoxon rank-sum test *p*-value = 6 x 10^-12^). Additionally, clips were enriched for residues with known catalytic roles from the Mechanism and Catalytic Site Atlas [70] (M-CSA) relative to other regions of the functional domain (odds ratio = 1.12, Fisher’s exact test *p*-value = 10^-34^). Together, these results indicate that local geometric distortion captures information relevant to protein stability and function.

**Fig 4:**
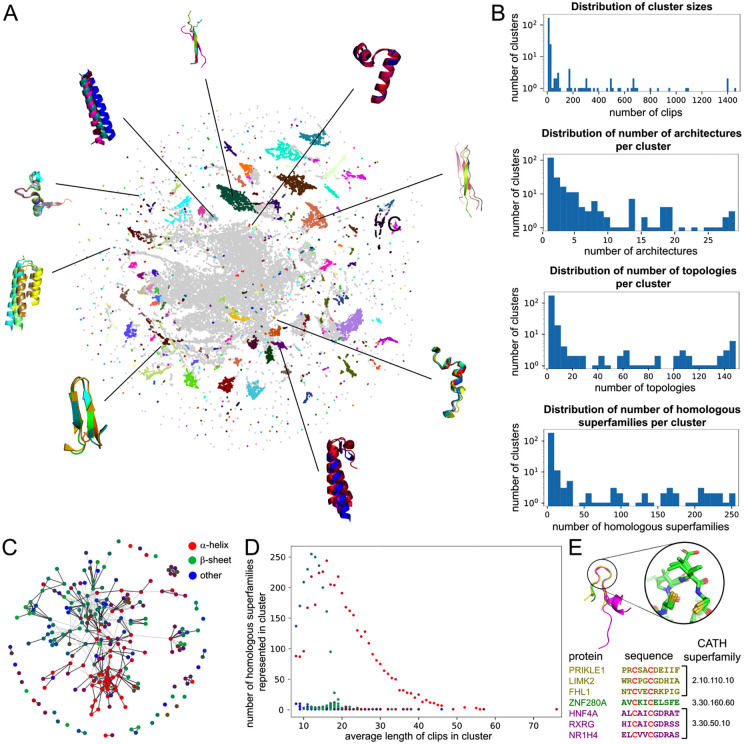
Structural motif analysis across human protein functional domains. **A)** UMAP embedding of structurally conserved protein short fragments across human functional domains. The space was clustered using DBSCAN, and clusters are shown in the UMAP, with representative fragments from selected clusters shown for reference. **B)** Distributions of the number of structural clips per cluster and the number of CATH architectures, topologies, and homologous superfamilies represented in each cluster. **C)** Network showing the co-occurrence of structural motifs within the same functional domain. Each node represents a cluster of structural clips, and edges represent significant co-occurrence (Fisher’s exact test FDR < 0.05) of two motifs within the same functional domain, with edge thickness proportional to the odds ratio (one-sided Fisher’s exact test). Nodes are colored by the proportion of residues in α-helices (red), β-sheets (green), or neither (blue). **D)** Number of CATH homologous superfamilies represented in each structural motif as a function of the average motif length. Points are colored by the secondary structure element composition, as in panel **(C)**. **E)** Examples of short clips belonging to the same structural motif (cluster ID 93), consisting of a small unstructured loop stabilized by a CXXC disulfide motif. The clips span seven proteins and functional domains from three different homologous superfamilies (represented with different colors). Protein names, amino acid sequences, and CATH homologous superfamily identifiers are also shown for reference.

To determine whether structural clips are unique to individual homologous domains or instead represent a shared structural lexicon across domain families, we computed pairwise GW distances between the 66,937 clips and clustered the resulting space using density-based clustering [71], yielding 234 clusters of structurally homologous clips, or “structural motifs” (Fig 4A, S7 Fig, and S3 Table). These structural motifs parallel the notion of recurrent local structural elements from previous works [65,72] but are derived from evolutionarily conserved subdomain units identified through alignment-based geometric distortions. Although each pairwise clip alignment took only a few milliseconds, the full analysis still required approximately 1 week on a standard 8-core desktop computer because of the very large number of pairwise comparisons (2.2 billion).

As expected, the space of clip structures was relatively continuous, and only 35% of the clips were assigned to a cluster by the density-based clustering algorithm. Each cluster contained an average of 101 clips (Fig 4B), and clip lengths within clusters were nearly unique, varying by at most three amino acids. Notably, each motif was found on average across 4.6 architectures, 18.8 topologies, and 27.7 homologous superfamilies of the CATH domain classification, indicating that these structural motifs are widely shared across the human proteome (Fig 4B). Moreover, pairs of structural motifs often significantly co-occurred within the same functional domain (Fig 4C; Fisher’s exact test FDR < 0.05), underscoring the combinatorial nature of the structural lexicon that underlies functional domains.

Visualizing the relationship between average clip length, number of homologous superfamilies, and proportion of residues in α-helices and β-sheets for each cluster revealed three main types of structural motifs (Fig 4D). The first type consists of motifs composed primarily of α-helices, often characterized by bends and diverse terminal loops. These span a wide range of lengths, with shorter motifs being more commonly observed across different CATH homologous superfamilies. The second type includes short motifs (< 20 residues) primarily composed of β-sheets, which are found in a large number (>75) of CATH homologous superfamilies. Finally, the third type consists of short motifs that appear in fewer than 25 homologous superfamilies. This type includes a wide variety of β-hairpin structures, as well as other structural motifs such as small loops stabilized by CXXC disulfide motifs (two cysteines separated by two other residues) (Fig 4E). Comparison with catalytic sites from M-CSA further revealed that some motifs identified in this analysis correspond to homologous structural fragments from catalytic sites with similar functional roles within the active site. For example, in cluster 14, which consists of 11-residue clips, residues with known catalytic roles all contribute to transition-state stabilization through hydrogen bonding across distinct catalytic sites (S3 Table).

Taken together, these results reveal a structural lexicon of small motifs shared across distinct functional domains, comprising not only α-helices and β-sheets but also other conserved small structural elements.

### Certain structural motifs show enrichment for pathogenic missense variants independent of domain context

To evaluate the functional relevance of short structural motifs independently of the functional domains in which they occur, we analyzed the positions of 11,130 missense single-nucleotide variants (SNVs) classified as pathogenic or likely pathogenic in the ClinVar database [73] across the 66,937 clips. These variants were more abundant in clips belonging to the 234 structural motifs (odds ratio = 1.26, Fisher’s exact *p*-value = 10^-24^ after adjusting for clip length).

When examining enrichments in individual motifs, we identified 10 structural motifs that were significantly enriched for pathogenic variants after accounting for clip length and CATH homologous superfamily membership as covariates (permutation test FDR < 0.05; S4 Table). While some of these enrichments were driven by specific proteins (FOXG1, LDLR, and RPGR) or small groups of closely related proteins (kinases, tubulin β-chains, UDP-glucuronosyltransferases, and cytochrome P450 enzymes), two structural motifs (cluster IDs 11 and 33) showed enrichment of pathogenic SNVs across structurally homologous clips from many unrelated proteins and homologous superfamilies. These motifs consisted of 12-amino-acid β-strand-like domain clips with repeated cysteine/histidine micro-motifs and 35-amino-acid membrane-associated amphipathic α-helices, respectively (S8 Fig), highlighting the broad structural and functional role of these motifs across the human proteome.

Collectively, these findings indicate that, consistent with the depletion of common variation in the structural motifs identified by GW correspondences, variants within these motifs are more likely to have pathogenic effects. Furthermore, the widespread occurrence of some of these structural motifs across the proteome provides a framework for relating pathogenic variants among otherwise seemingly unrelated proteins and functional domains.

## Discussion

Metric geometry is the branch of mathematics concerned with the study and comparison of shapes. Here, we investigated the application of metric geometry, and in particular GW correspondences, to the comparative study of protein structures.

Several aspects make GW correspondences particularly attractive in this context. Being mathematically well-grounded, several theoretical results [28–30] ensure convergence, optimality, stability, and other desirable properties, including the definition of true distances, which facilitate robust implementation of downstream analyses and approximations. For example, the triangle inequality imposes constraints on structural pairwise distances that have not yet been computed, allowing one to determine whether their computation is necessary. This can substantially reduce computational cost, since in most applications only distances to the nearest neighbors need to be computed accurately [34]. Moreover, the modular nature of the GW framework provides flexibility that can be tailored to specific applications. For instance, fused versions of GW correspondences [45] enable the integration of biochemical information into structural alignments, such as isoelectric points, hydrophobicity, or BLOSUM scores. GW correspondences can also be interpreted locally, allowing the identification of structurally conserved regions. In addition, because they do not rely on sequence information, they remain robust to topologically non-trivial sequence rearrangements.

We have demonstrated these properties across various applications, including the analysis of switch regions in the KRAS oncoprotein, the identification of structurally conserved catalytic sites in RdRps, and the classification of short structural motifs in the human proteome. Our results demonstrate the utility and potential of metric geometry, offering comparable, and in some cases superior, results to existing methods, thereby positioning it as a useful and complementary framework for comparative protein structure analyses. Given the maturity of existing methods, built on decades of algorithmic development and optimization, and the rapid evolution of the emerging discipline of applied metric geometry, with extensions and algorithms for computing GW correspondences advancing quickly, we envision metric geometry as a promising new framework for comparative protein structure analyses whose impact will continue to grow in the years ahead.

Our work has also revealed current limitations in the application of metric geometry to protein structure analysis. In particular, balanced GW correspondences such as those used here, which do not allow partial matchings, pose challenges when comparing proteins that differ substantially in size or contain insertions or deletions of small fragments. Although unbalanced and partial GW formulations could in principle address this limitation [40,46,74], their practical application to protein structure alignment remains challenging. Their higher computational cost, together with sensitivity to parameter choices and complex optimization landscape, currently limits their routine use in this setting. We anticipate that continued algorithmic advances in applied metric geometry will help overcome these challenges and broaden the applicability of GW-based approaches to comparative protein structure analysis.

## Methods

### Protein structural alignment with Gromov-Wasserstein correspondences

We define a correspondence between proteins X and Y as a n×m matrix T with entries $T_{i,k}$ in the interval [0,1] such that $\sum_{i}T_{i,k}=\frac{\text{1}}{m}$ and $\sum_{k}T_{i,k}=\frac{\text{1}}{n}$, where n and m are the number of residues in X and Y, respectively. Thus, T can be interpreted as a probability distribution supported on an n×m grid with uniform marginals.

Given a correspondence T, we define its GW cost as


$$\begin{array}{c} \sum_{i,j,k,l}{\left|d\left(x_{i},x_{j}\right)-d\left(y_{k},y_{l}\right)\right|}^{\text{2}}T_{i,k}T_{j,l} \end{array}$$ (1)

where $d\left(x_{i},x_{j}\right)\;$and $d\left(y_{k},y_{l}\right)\;$are Euclidean distances between α-carbons $x_{i}$ and $x_{j}$ in X, and $y_{k}$ and $y_{l}$ in Y, respectively. The GW distance between X and Y is then given by [29]


$$\begin{array}{c} GW(X,Y)=\;\frac{\text{1}}{\text{2}}\underset{T}{\text{min}}{\left(\sum_{i,j,k,l}{\left|d\left(x_{i},x_{j}\right)-d\left(y_{k},y_{l}\right)\right|}^{\text{2}}T_{i,k}T_{j,l}\right)}^{\frac{\text{1}}{\text{2}}} \end{array}$$ (2)

where the minimum is taken over the space of possible correspondences. This quantity satisfies the axioms of a metric space [29].

Let $\delta \left(x_{i},y_{k}\right)$ be a non-negative, symmetric function that quantifies the difference between the biochemical properties of any two amino acids. This function can represent a difference in scalar features like isoelectric point, hydrophobicity, or solvent accessible surface area; or pairwise measures, such as the Grantham distance or BLOSUM-based distances. We define the fused GW (FGW) distance as [45]


$$\begin{array}{c} FGW(X,Y)=\;\frac{\text{1}}{\text{2}}\underset{T}{\text{min}}{\left(\sum_{i,j,k,l}(\;\alpha {\cdot \;\left|d\left(x_{i},x_{j}\right)-d\left(y_{k},y_{l}\right)\right|}^{\text{2}}{+\;(\text{1}-\alpha )\cdot \delta \left(x_{i},y_{k}\right)\;)\;T}_{i,k}T_{j,l}\right)}^{\frac{\text{1}}{\text{2}}} \end{array}$$ (3)

where the parameter α∈[0,1] controls the relative contribution of the geometric and biochemical distortions. Setting α=1 recovers the standard GW distance, while α=0 corresponds to alignment based solely on biochemical data, ignoring geometric constraints. If δ defines a metric, then the FGW also defines a metric for a fixed value of α.

To solve the non-convex optimization problems in Eqs. (2) and (3), we use the Frank-Wolfe (conditional gradient) method described by Vayer *et al.*[75], as implemented in the Python Optimal Transport (POT) library [76] and the CAJAL package [34]. In this approach, the inner oracle for the linearized subproblem is solved using the network simplex algorithm. Unless otherwise noted, the optimization was initialized with the identity map.

Given a correspondence T between two protein structures X and Y, we define the local geometric distortion of a C_α_ atom in X as its contribution to the GW cost function. Specifically,


$$\begin{array}{c} D\left(x_{\text{0}}\right)=\sum_{j,k,l}{\left|d_{X}\left(x_{\text{0}},x_{j}\right)-d_{Y}\left(y_{k},y_{l}\right)\right|}^{\text{2}}T_{\text{0},k}T_{j,l} \end{array}$$ (4)

It follows that $\sum_{i}D\left(x_{i}\right)=\;\sum_{k}D(y_{k})=\text{4}\;GW(X,Y{)}^{\text{2}}$. Similarly, in the case of FGW, we define


$$\begin{array}{c} FD\left(x_{\text{0}}\right)=\sum_{j,k,l}(\;\alpha {\cdot \;\left|d\left(x_{\text{0}},x_{j}\right)-d\left(y_{k},y_{l}\right)\right|}^{\text{2}}{+\;(\text{1}-\alpha )\cdot \delta \left(x_{\text{0}},y_{k}\right)\;)\;T}_{\text{0},k}T_{j,l} \end{array}$$ (5)

and $\sum_{i}FD\left(x_{i}\right)=\;\sum_{k}FD(y_{k})=\text{4}\;FGW(X,Y{)}^{\text{2}}$. To the best of our knowledge, this pointwise use of GW costs to quantify the geometric distortion of a point $x_{\text{0}}$ when mapped to Y via a correspondence T is novel.

When comparing N protein structures in an all-vs-all fashion to identify local geometric distortions, we first perform all N(N−1)/2 pairwise GW (or FGW) computations. For each pair of structures, we store the GW distance and correspondence, and the local geometric distortions.

For a given protein structure, each C_α_ atom accumulates N−1 local geometric distortion values (one from each comparison with the other protein structures). We average these values (in some cases, using a weighted average, as described below) and then transfer the averages across the protein structures using the GW correspondences. Explicitly, the transferred average local geometric distortion for C_α_ atom $x_{i}$ in X from Y is given by $\sum_{k}T_{i,k}\overset{―}{D}(y_{k})$, where T is the correspondence between X and Y, and $\overset{―}{D}(y_{k})$ is the averaged local geometric distortion for C_α_ atom $y_{k}$ in protein Y. This yields N−1 transferred average local distortions per C_α_ atom, which can again be aggregated. This double-averaging procedure substantially enhances the ability to capture local structural variation across the ensemble of protein structures by incorporating composed correspondences.

For visualization, we normalize the local or average local geometric distortion within each protein structure such that the minimum value is 0 and the maximum is 1.

### Hyperparameter selection

The main hyperparameters in our analyses were α and the number of sampled residues. To choose α, we examined the relative magnitudes of the two terms in the FGW objective function (Eq. (3)) and identified a range of values for which the two contributions were comparable. We then verified that the quantitative performance metrics (MCC, AUPRC) remained relatively stable across this range and visually inspected a small subset of alignments as a sanity check.

The number of sampled residues was chosen based on computational constraints: we used the largest value that could be run within a reasonable time on a standard 8-core Intel Xeon desktop computer.

Analyses that use local geometric distortion to identify structurally conserved regions also depended on the threshold chosen for this quantity. This threshold controls the tradeoff between type I and type II errors. Whenever feasible, we varied it to construct precision-recall curves; otherwise, we used the median value as a conservative default.

### Identification of KRAS switch regions

We downloaded the PDB files of 54 KRAS protein structures from the RCSB Protein Data Bank [8], selecting only those determined by X-ray crystallography and containing at most 3 non-synonymous single-nucleotide variants relative to the UniProt [77] canonical sequence (accession P01116-1) within residues 2–162 (S1 Table). We restricted our analysis to this sequence region. For PDB files containing multiple KRAS chains, each chain was extracted and saved as a separate structure.

As there is no consensus on the exact boundaries of the switch regions [47], we adopted residues 30–40 for switch I and 60–72 for switch II as our working definitions.

We aligned all structures using GWProt and computed the average local geometric distortion for each C_α_ atom, as described in subsection “*Protein structural alignment with Gromov-Wasserstein correspondences*” of the Methods section. We computed a single average local geometric distortion for each C_α_ atom, without transferring across structures and second averaging, since the high structural homology made this unnecessary.

To evaluate the predictive power of the averaged local geometric distortion in identifying switch regions, we treated regions with distortion levels exceeding a threshold as predicted switch sites, and calculated precision-recall curves for each protein by varying this threshold.

### Identification of functional sites in RdRps

We analyzed computationally predicted structures of the core domains of 2,777 riboviral RdRps with amino acid sequences deposited in GenPept (S2 Table), of which 2,739 belonged to one of 14 classes containing at least 10 representatives. Structures were predicted from sequences using AlphaFold [10] and trimmed to a region of ~500 residues corresponding to the central core of the RdRp domain. These structures were drawn from a curated structural benchmark of viral and non-viral palm-domain structures assembled by R. C. Edgar in connection with earlier work [48,49] (R. C. Edgar, personal communication). This benchmark comprises SCOP-annotated palm-domain PDB structures and selected high-quality AlphaFold models (based on pLDDT score) of GenBank proteins identified as close homologs by BLAST- or HMM-based searches, including both canonical and permuted palm-domain architectures.

We computed pairwise GW correspondences between the RdRps using GWProt, as described in subsection “*Protein structural alignment with Gromov-Wasserstein correspondences*” of the Methods section. To reduce computation time given the large number of pairwise comparisons (~3.8 million), we downsampled each structure to 200 evenly spaced residues.

Additionally, we computed pairwise GW correspondences without downsampling for 97 randomly selected RdRps from the same dataset. For these proteins, we identified the locations of the A, B, and C regions using Palmscan [48] with default parameters, and visually confirmed them in PyMOL. We use the GW correspondences to compute local geometric distortion using the double averaging approach described in subsection “*Protein structural alignment with Gromov-Wasserstein correspondences*”. To assess the predictive power of the averaged local geometric distortion for identifying A, B, and C sites, we treated regions with distortion levels below a threshold as predicted A, B, or C sites, and calculated precision-recall curves for each protein by varying this threshold. We compared this with a baseline in which each pair of structures was superposed using RMSD-minimizing rigid-body alignment with the Kabsch algorithm [63], as implemented in PyMOL’s ‘align’ command, and residue-level deviations were used in place of local geometric distortion to predict A, B, and C sites. Unaligned residues were set to the maximum deviation observed between the pair of structures.

Finally, we repeated the analysis on the same set of 97 RdRps using FGW correspondences instead of GW, with parameter α=0.2 and δ defined as the difference between the standardized consensus hydrophobicity level of the two residues [78].

### Evaluation of GW distances for the identification of novel RdRps

We considered the computationally predicted folds of the core domains of 150 randomly selected RdRps from each riboviral phylum (Duplornaviricota, Kitrinoviricota, Lenarviricota, Negarnaviricota, and Pisuviricota) with sequence lengths between 200 and 700 residues (S2 Table). For Lenarviricota, only 124 predicted folds were available, and all were included. We augmented this dataset with 300 decoy structures consisting of close non-RdRp palm-domain homologs from the same curated benchmark described in subsection “*Identification of functional sites in RdRps*”.

We constructed a protein structure embedding space for this combined dataset using FGW, with parameter α=0.2, δ defined as the difference between the standardized consensus hydrophobicity level of the two residues [78], and inter-carbon distances scaled using the square root function. Similar embedding spaces were also generated using TM-align [5], with parameters -fast -a T, Foldseek [6], with parameters easy-search –-exhaustive-search 1, and RMSD-minimizing rigid-body alignment, implemented in PyMOL,

For each phylum, we then trained a *k* = 3 nearest-neighbor classifier on each of the three embedding spaces using 10-fold cross-validation. In each iteration, 10% of the decoys and 10% of the RdRps from the focal phylum were used as test data, while RdRps from other phyla and the remaining decoys served as the training set. The accuracy of the classifier was quantified for each phylum and embedding space using the MCC.

To evaluate runtime as a function of protein length, we randomly sampled pairs of RdRps and selected polypeptide chains of length n, ranging from 10 to 1,000 residues. We then ran GWProt in a single thread on 1,000 such polypeptide-chain pairs and used log-log linearization to estimate the power law governing runtime in seconds.

### Analysis of structural motifs in human protein functional domains

We considered all homologous superfamilies in the CATH database that contained at least 5 human protein domains and obtained their computationally predicted structures from AlphaFold2 as reported by Bordin et al. [79]. For the 78 superfamilies containing over 50 domains, we randomly selected 50 structures. For each domain, we removed residues at the domain termini with predicted Local Distance Difference Test (pLDDT) scores ≤ 75 and excluded domains with fewer than 20 remaining amino acids. After applying these filters, 10,139 computationally folded domains remained.

We computed all pairwise GW correspondences between domains within each homologous superfamily and averaged the local geometric distortions, following the procedure described in the subsection “*Protein structural alignment with Gromov-Wasserstein correspondences*”. To emphasize residues with high local geometric distortion, while keeping distortion values independent of protein size or the number of domains in a superfamily, we averaged the squared distortion values, weighting each domain by the cube of its length and inversely by the total sum of distortion values, and then averaged the transferred distortion values weighted by the length of the domain.

We then computed a rolling average of the local geometric distortion values using a 7-residue window, and selected residues that had an average distortion below the median distortion for the domain (and at most 100,000), except for residues whose average distortion exceeded that of the neighboring 8 residues on each side.

Contiguous residues were grouped into clips. Clips shorter than 8 residues were discarded unless they were within two residues of another clip, in which case the two clips were merged into a single clip. The resulting 66,937 clips were clustered according to their GW distance using the density-based algorithm DBSCAN [71], as implemented in the Python package scikit-learn, with parameters leaf_size = 100 and min_samples = 10, yielding 23,668 clips grouped into 234 clusters. For each cluster, the proportion of amino acids identified as belonging to α-helices and β-sheets was inferred using the algorithm STRIDE [80].

For each pair of clusters, we counted the number of domains containing clips from both clusters, from only one of the clusters, or from neither. We then performed a one-sided Fisher’s exact test and corrected for multiple hypothesis testing using the Benjamini-Hochberg procedure. Finally, we constructed an adjacency graph of co-occurrences with q-values < 0.05, weighting the edges by the corresponding odds ratios.

For the analysis of common variation, we annotated common SNPs in the 10,139 CATH domains using SnpEff v5.2f [81] and the dbSNP database [82] (build 151), identifying a total of 19,059 residues with common missense SNPs. We then counted the number of residues within clips and outside clips (but still within domains), both with and without common missense SNPs, and performed a Fisher’s exact test to assess the significance of the association between clips and common variants.

Similarly, in the analysis of catalytic sites, we used the M-CSA database [70], including both reference proteins and homologs, to annotate CATH domain residues with known catalytic roles, yielding a total of 1,228 annotated residues. We then followed the same procedure as in the SNP analysis to assess the significance of the association between clips and catalytic sites.

To evaluate the stability of structural clips, we calculated the average normalized fitness score for each residue in 341 domains from 203 proteins that overlapped with the site-saturation mutagenesis screen of Beltran et al. [69]. We then assessed the significance of the difference in mean normalized fitness scores between residues in clips and those outside clips (but still within domains) using the Wilcoxon rank-sum test.

### Analysis of pathogenic variants within structural motifs

We retrieved all pathogenic and likely pathogenic missense SNVs from the ClinVar database [73] (update 2025-08-31) and annotated their position in the clips using SnpEff v5.2f[81]. In total, 11,130 clip positions contained at least one pathogenic or likely pathogenic SNV.

For hypothesis testing, we used the total number of SNV locations within each cluster as our test statistic. We tested clusters with at least 4 SNV locations using a permutation test with 10,000 random permutations. In each permutation, and for each CATH homologous superfamily, we randomly reassigned SNV locations among all clips belonging to domains from that superfamily, with the probability of relocation to a clip proportional to its length. For each cluster, we then calculated the fraction of permutations in which the total number of SNV locations was at least as high as in the unpermuted one. We controlled the false discovery rate using the Benjamini-Hochberg procedure.

## Supporting information

S1 Fig Identification of structurally conserved catalytic sites in RdRps using GW correspondences. **A)** Example of an RdRp core domain from Hepacivirus hominis (GenPept ID AFD18577) colored by average GW local geometric distortion. Regions of low distortion, mostly located at the catalytic core, are structurally conserved. **B)** Distribution of Spearman correlation coefficients between local geometric distortion and residue-level deviations from RMSD-minimizing rigid-body alignment for each RdRp core domain in each pairwise alignment. **C)** Median, 20%, and 80% percentile precision-recall curves for predicting A, B, or C sites based on average GW local geometric distortion (blue) and average residue-level deviations from RMSD-minimizing rigid-body alignment (red) across 97 randomly selected RdRps. AUPRC: area under the precision-recall curve; RMSD: root-mean-square deviation.
(TIFF)

S2 FigStability analysis of GW correspondences and local geometric distortion.For each of 50 randomly selected pairs of RdRps, we compared the GW correspondence and local geometric distortions obtained from the default initialization $P_{\text{0}}$ with those obtained from initializations of the form $aP_{r}+(\text{1}-a)P_{\text{0}}$, where a∈[0,1] denotes the proportion of randomness and $P_{r}$ is a random initial alignment generated by uniform sampling from the space of feasible correspondences using hit-and-run Markov chain Monte Carlo. For reference, the GW correspondences were also compared with random correspondences.(TIFF)

S3 Fig Dot plot visualization of the GW (black) and MultiProt (red) correspondences between two RdRp core domains with ABC and CAB active site orderings. (TIFF)

S4 FigIdentification of RdRp core domain structures from previously unseen phyla.**A)** Shown are the average 10-fold cross-validation MCC values and standard deviations (in parenthesis) for a *k* = 3 nearest neighbor classifier trained on structural embedding spaces produced by GWProt, TM-align, Foldseek, and rigid-body alignment minimizing RMSD. The classifiers were evaluated on the task of distinguishing RdRp core domain structures belonging to phyla absent from the training data from non-RdRp decoys with high amino acid sequence similarity to bona fide RdRp core domains. The MCC of a majority-vote classifier combining GWProt, TM-align, and Foldseek is also shown. Runtimes on a standard 8-core desktop computer using parallelization are also indicated. **B)** Average 10-fold cross-validation MCC values and standard deviations are shown for GWProt without downsampling and with regular downsampling to 200, 100, and 50 residues. Runtimes on a standard 8-core desktop computer using parallelization are also indicated. **C)** Average 10-fold cross-validation MCC values and standard deviations are shown for GWProt applied to 100 regularly downsampled residues with different shifts (Δ=−50%, 0%, +50% of the distance between sampled residues) and to 100 randomly sampled residues with equal probability.(TIFF)

S5 FigRuntime of GWProt as a function of protein length.Runtime of GWProt, in seconds, as a function of the number of residues for 1,000 randomly sampled RdRp polypeptide-chain pairs. The fitted line follows the empirical scaling law $t≃\text{7}\times {\text{1}\text{0}}^{-\text{7}}\;n^{\text{2}.\text{3}}$.(TIFF)

S6 Fig Effect of clipping algorithm parameters on clip length and number of clips per domain. The algorithm was applied to 50 randomly selected homologous superfamilies (1,667 domains total) across varying rolling-window sizes (keeping the threshold on average geometric distortion fixed at 50%) and thresholds on average local geometric distortion (keeping the window size fixed at 7 residues).
(TIFF)

S7 Fig UMAP embedding of the structural space of domain clips, colored according to secondary structure element composition. (TIFF)

S8 Fig Representative clips from structural motifs 11 and 33. Positions with known pathogenic or likely pathogenic SNVs are indicated in red.
(TIFF)

S1 Table RCSB Protein Data Bank accession numbers and metadata of the 54 KRAS protein crystallographic structures included in the analysis. (CSV)

S2 Table GenPept accession numbers and taxonomy of the RdRps and non-RdRp decoys included in the analysis. (CSV)

S3 Table List of structurally conserved polypeptides across human functional protein domains. (CSV)

S4 Table Pathogenic missense variant enrichment in structural motifs. (CSV)

## References

[1] Hasegawa H, Holm L. Advances and pitfalls of protein structural alignment. Curr Opin Struct Biol. 2009;19(3):341–8. doi: 10.1016/j.sbi.2009.04.003 19481444

[2] Holm L, Sander C. Dali: A network tool for protein structure comparison. Trends Biochem Sci. 1995;20(11):478–80. doi: 10.1016/s0968-0004(00)89105-7 8578593

[3] Orengo CA, Taylor WR. SSAP: Sequential structure alignment program for protein structure comparison. Methods Enzymol. 1996;266:617–35. doi: 10.1016/s0076-6879(96)66038-8 8743709

[4] Shindyalov IN, Bourne PE. Protein structure alignment by incremental combinatorial extension (CE) of the optimal path. Protein Eng. 1998;11(9):739–47. doi: 10.1093/protein/11.9.739 9796821

[5] Zhang Y, Skolnick J. TM-align: A protein structure alignment algorithm based on the TM-score. Nucleic Acids Res. 2005;33(7):2302–9. doi: 10.1093/nar/gki524 15849316 PMC1084323

[6] van Kempen M, Kim SS, Tumescheit C, Mirdita M, Lee J, Gilchrist CLM, et al. Fast and accurate protein structure search with Foldseek. Nat Biotechnol. 2024;42(2):243–6. doi: 10.1038/s41587-023-01773-0 37156916 PMC10869269

[7] Edgar RC. Protein structure alignment by Reseek improves sensitivity to remote homologs. Bioinformatics. 2024;40(11):btae687. doi: 10.1093/bioinformatics/btae687 39546374 PMC11601161

[8] Berman HM, Westbrook J, Feng Z, Gilliland G, Bhat TN, Weissig H, et al. The protein data bank. Nucleic Acids Res. 2000;28(1):235–42. doi: 10.1093/nar/28.1.235 10592235 PMC102472

[9] Baek M, DiMaio F, Anishchenko I, Dauparas J, Ovchinnikov S, Lee GR, et al. Accurate prediction of protein structures and interactions using a three-track neural network. Science. 2021;373(6557):871–6. doi: 10.1126/science.abj8754 34282049 PMC7612213

[10] Jumper J, Evans R, Pritzel A, Green T, Figurnov M, Ronneberger O, et al. Highly accurate protein structure prediction with AlphaFold. Nature. 2021;596(7873):583–9. doi: 10.1038/s41586-021-03819-2 34265844 PMC8371605

[11] Lin Z, Akin H, Rao R, Hie B, Zhu Z, Lu W, et al. Evolutionary-scale prediction of atomic-level protein structure with a language model. Science. 2023;379(6637):1123–30. doi: 10.1126/science.ade2574 36927031

[12] Orengo CA, et al. CATH--a hierarchic classification of protein domain structures. Structure. 1997;5:1093–108. doi: 10.1016/s0969-2126(97)00260-8 9309224

[13] Murzin AG, Brenner SE, Hubbard T, Chothia C. SCOP: A structural classification of proteins database for the investigation of sequences and structures. J Mol Biol. 1995;247(4):536–40. doi: 10.1006/jmbi.1995.0159 7723011

[14] Sonnhammer EL, Eddy SR, Durbin R. Pfam: A comprehensive database of protein domain families based on seed alignments. Proteins. 1997;28(3):405–20. doi: 10.1002/(sici)1097-0134(199707)28:3<405::aid-prot10>3.0.co;2-l 9223186

[15] Lau AM, Bordin N, Kandathil SM, Sillitoe I, Waman VP, Wells J, et al. Exploring structural diversity across the protein universe with The Encyclopedia of Domains. Science. 2024;386(6721):eadq4946. doi: 10.1126/science.adq4946 39480926 PMC7618865

[16] Durairaj J, Waterhouse AM, Mets T, Brodiazhenko T, Abdullah M, Studer G, et al. Uncovering new families and folds in the natural protein universe. Nature. 2023;622(7983):646–53. doi: 10.1038/s41586-023-06622-3 37704037 PMC10584680

[17] James Milner-White E, Poet R. Loops, bulges, turns and hairpins in proteins. Trends in Biochemical Sciences. 1987;12:189–92. doi: 10.1016/0968-0004(87)90091-0

[18] Fernandez-Fuentes N, Oliva B, Fiser A. A supersecondary structure library and search algorithm for modeling loops in protein structures. Nucleic Acids Res. 2006;34(7):2085–97. doi: 10.1093/nar/gkl156 16617149 PMC1440879

[19] Richardson JS. The anatomy and taxonomy of protein structure. Adv Protein Chem. 1981;34:167–339. doi: 10.1016/s0065-3233(08)60520-3 7020376

[20] Kolodny R, Koehl P, Guibas L, Levitt M. Small libraries of protein fragments model native protein structures accurately. J Mol Biol. 2002;323(2):297–307. doi: 10.1016/s0022-2836(02)00942-7 12381322

[21] Unger R, Harel D, Wherland S, Sussman JL. A 3D building blocks approach to analyzing and predicting structure of proteins. Proteins. 1989;5(4):355–73. doi: 10.1002/prot.340050410 2798411

[22] Derry A, Altman RB. COLLAPSE: A representation learning framework for identification and characterization of protein structural sites. Protein Sci. 2023;32(2):e4541. doi: 10.1002/pro.4541 36519247 PMC9847082

[23] Derry A, Krupkin H, Tartici A, Altman RB. Unsupervised learning reveals landscape of local structural motifs across protein classes. Bioinformatics. 2025;41(7):btaf377. doi: 10.1093/bioinformatics/btaf377 40569048 PMC12258146

[24] Wu S, Liu T, Altman RB. Identification of recurring protein structure microenvironments and discovery of novel functional sites around CYS residues. BMC Struct Biol. 2010;10:4. doi: 10.1186/1472-6807-10-4 20122268 PMC2833161

[25] Burago D, Burago Y, Ivanov SA. A course in metric geometry. Providence: American Mathematical Society. 2001.

[26] Fréchet MM. Sur quelques points du calcul fonctionnel. Rendiconti del Circolo Matematico di Palermo. 1906;22:1–72.

[27] Gromov M. Metric structures for Riemannian and non-Riemannian spaces. Springer Science & Business Media. 2007.

[28] Mémoli F. On the use of Gromov-Hausdorff distances for shape comparison. 2007.

[29] Mémoli FG. Gromov–Wasserstein distances and the metric approach to object matching. Foundations of Computational Mathematics. 2011;11:417–87.

[30] Mémoli F, Sapiro G. A theoretical and computational framework for isometry invariant recognition of point cloud data. Found Comput Math. 2005;5(3):313–47. doi: 10.1007/s10208-004-0145-y

[31] Titouan V, Flamary R, Courty N, Tavenard R, Chapel L. Sliced gromov-wasserstein. Advances in Neural Information Processing Systems. 2019;32.

[32] Chowdhury S, Miller D, Needham T. Springer International Publishing. 811–27.

[33] Scetbon M, Peyré G, Cuturi M. International Conference on Machine Learning. 19347–65.

[34] Govek KW, Nicodemus P, Lin Y, Crawford J, Saturnino AB, Cui H, et al. CAJAL enables analysis and integration of single-cell morphological data using metric geometry. Nat Commun. 2023;14(1):3672. doi: 10.1038/s41467-023-39424-2 37339989 PMC10282047

[35] Demetci P, Santorella R, Chakravarthy M, Sandstede B, Singh R. SCOTv2: Single-cell multiomic alignment with disproportionate cell-type representation. J Comput Biol. 2022;29(11):1213–28. doi: 10.1089/cmb.2022.0270 36251763 PMC9805876

[36] Demetci P, Santorella R, Sandstede B, Noble WS, Singh R. SCOT: Single-cell multi-omics alignment with optimal transport. J Comput Biol. 2022;29(1):3–18. doi: 10.1089/cmb.2021.0446 35050714 PMC8812493

[37] Cao K, Hong Y, Wan L. Manifold alignment for heterogeneous single-cell multi-omics data integration using Pamona. Bioinformatics. 2021;38(1):211–9. doi: 10.1093/bioinformatics/btab594 34398192 PMC8696097

[38] Liu X, Zeira R, Raphael BJ. Partial alignment of multislice spatially resolved transcriptomics data. Genome Res. 2023;33(7):1124–32. doi: 10.1101/gr.277670.123 37553263 PMC10538490

[39] Zeira R, Land M, Strzalkowski A, Raphael BJ. Alignment and integration of spatial transcriptomics data. Nat Methods. 2022;19(5):567–75. doi: 10.1038/s41592-022-01459-6 35577957 PMC9334025

[40] Thual A, et al. Aligning individual brains with fused unbalanced Gromov Wasserstein. Advances in Neural Information Processing Systems. 2022;35:21792–804.

[41] Xu H, Luo D, Zha H, Duke LC. In: International conference on machine learning. 6932–41.

[42] Cuturi M. Sinkhorn distances: Lightspeed computation of optimal transport. Advances in neural information processing systems. 2013;26.

[43] Peyré G, Cuturi M, Solomon J. International Conference on Machine Learning. 2664–72.

[44] Scetbon M, Cuturi M, Peyré G. International Conference on Machine Learning. 9344–54. PMLR.

[45] Vayer T, Chapel L, Flamary R, Tavenard R, Courty N. Fused gromov-wasserstein distance for structured objects. Algorithms. 2020;13(9):212. doi: 10.3390/a13090212

[46] Séjourné T, Vialard FX, Peyré G. The unbalanced gromov wasserstein distance: Conic formulation and relaxation. Advances in Neural Information Processing Systems. 2021;34:8766–79.

[47] Pantsar T. The current understanding of KRAS protein structure and dynamics. Comput Struct Biotechnol J. 2019;18:189–98. doi: 10.1016/j.csbj.2019.12.004 31988705 PMC6965201

[48] Babaian A, Edgar R. Ribovirus classification by a polymerase barcode sequence. PeerJ. 2022;10:e14055. doi: 10.7717/peerj.14055 36258794 PMC9573346

[49] Edgar RC, Taylor B, Lin V, Altman T, Barbera P, Meleshko D, et al. Petabase-scale sequence alignment catalyses viral discovery. Nature. 2022;602(7895):142–7. doi: 10.1038/s41586-021-04332-2 35082445

[50] Wolf YI, et al. Origins and evolution of the global RNA virome. mBio. 2018;9. doi: 10.1128/mBio.02329-18 PMC6282212 30482837

[51] Mönttinen HAM, Ravantti JJ, Stuart DI, Poranen MM. Automated structural comparisons clarify the phylogeny of the right-hand-shaped polymerases. Mol Biol Evol. 2014;31(10):2741–52. doi: 10.1093/molbev/msu219 25063440

[52] te Velthuis AJW. Common and unique features of viral RNA-dependent polymerases. Cell Mol Life Sci. 2014;71(22):4403–20. doi: 10.1007/s00018-014-1695-z 25080879 PMC4207942

[53] Bruenn JA. A structural and primary sequence comparison of the viral RNA-dependent RNA polymerases. Nucleic Acids Res. 2003;31(7):1821–9. doi: 10.1093/nar/gkg277 12654997 PMC152793

[54] McInnes L, Healy J, Saul N, Großberger L. UMAP: Uniform manifold approximation and projection. JOSS. 2018;3(29):861. doi: 10.21105/joss.00861

[55] Bliven S, Prlić A. Circular permutation in proteins. PLoS Comput Biol. 2012;8(3):e1002445. doi: 10.1371/journal.pcbi.1002445 22496628 PMC3320104

[56] Gorbalenya AE, Pringle FM, Zeddam J-L, Luke BT, Cameron CE, Kalmakoff J, et al. The palm subdomain-based active site is internally permuted in viral RNA-dependent RNA polymerases of an ancient lineage. J Mol Biol. 2002;324(1):47–62. doi: 10.1016/s0022-2836(02)01033-1 12421558 PMC7127740

[57] Sabanadzovic S, Ghanem-Sabanadzovic NA, Gorbalenya AE. Permutation of the active site of putative RNA-dependent RNA polymerase in a newly identified species of plant alpha-like virus. Virology. 2009;394(1):1–7. doi: 10.1016/j.virol.2009.08.006 19793602

[58] Bliven SE, Bourne PE, Prlić A. Detection of circular permutations within protein structures using CE-CP. Bioinformatics. 2015;31(8):1316–8. doi: 10.1093/bioinformatics/btu823 25505094 PMC4393524

[59] Bachar O, Fischer D, Nussinov R, Wolfson H. A computer vision based technique for 3-D sequence-independent structural comparison of proteins. Protein Eng. 1993;6(3):279–88. doi: 10.1093/protein/6.3.279 8506262

[60] Leibowitz N, Nussinov R, Wolfson HJ. MUSTA--a general, efficient, automated method for multiple structure alignment and detection of common motifs: application to proteins. J Comput Biol. 2001;8(2):93–121. doi: 10.1089/106652701300312896 11454300

[61] Shatsky M, Nussinov R, Wolfson HJ. A method for simultaneous alignment of multiple protein structures. Proteins. 2004;56(1):143–56. doi: 10.1002/prot.10628 15162494

[62] Dror O, Benyamini H, Nussinov R, Wolfson HJ. Multiple structural alignment by secondary structures: Algorithm and applications. Protein Sci. 2003;12(11):2492–507. doi: 10.1110/ps.03200603 14573862 PMC2366961

[63] Kabsch W. A solution for the best rotation to relate two sets of vectors. Foundations of Crystallography. 1976;32:922–3.

[64] de Brevern AG, Valadié H, Hazout S, Etchebest C. Extension of a local backbone description using a structural alphabet: A new approach to the sequence-structure relationship. Protein Sci. 2002;11(12):2871–86. doi: 10.1110/ps.0220502 12441385 PMC2373739

[65] Mackenzie CO, Zhou J, Grigoryan G. Tertiary alphabet for the observable protein structural universe. Proc Natl Acad Sci U S A. 2016;113(47):E7438–47. doi: 10.1073/pnas.1607178113 27810958 PMC5127300

[66] Nepomnyachiy S, Ben-Tal N, Kolodny R. Global view of the protein universe. Proc Natl Acad Sci U S A. 2014;111(32):11691–6. doi: 10.1073/pnas.1403395111 25071170 PMC4136566

[67] Simons KT, Kooperberg C, Huang E, Baker D. Assembly of protein tertiary structures from fragments with similar local sequences using simulated annealing and Bayesian scoring functions. J Mol Biol. 1997;268(1):209–25. doi: 10.1006/jmbi.1997.0959 9149153

[68] Gront D, Kulp DW, Vernon RM, Strauss CEM, Baker D. Generalized fragment picking in Rosetta: Design, protocols and applications. PLoS One. 2011;6(8):e23294. doi: 10.1371/journal.pone.0023294 21887241 PMC3160850

[69] Beltran A, Jiang X, Shen Y, Lehner B. Site-saturation mutagenesis of 500 human protein domains. Nature. 2025;637(8047):885–94. doi: 10.1038/s41586-024-08370-4 39779847 PMC11754108

[70] Ribeiro AJM, et al. Mechanism and Catalytic Site Atlas (M-CSA): A database of enzyme reaction mechanisms and active sites. Nucleic Acids Research. 2018;46:D618–23. doi: 10.1093/nar/gkx1012 PMC5753290 29106569

[71] Ester M, Kriegel HP, Sander J, Xu X. KDD. 226–31.

[72] Alva V, Söding J, Lupas AN. A vocabulary of ancient peptides at the origin of folded proteins. Elife. 2015;4:e09410. doi: 10.7554/eLife.09410 26653858 PMC4739770

[73] Landrum MJ, Lee JM, Riley GR, Jang W, Rubinstein WS, Church DM, et al. ClinVar: public archive of relationships among sequence variation and human phenotype. Nucleic Acids Res. 2014;42(Database issue):D980-5. doi: 10.1093/nar/gkt1113 24234437 PMC3965032

[74] Chapel L, Alaya MZ, Gasso G. Partial optimal transport with applications on positive-unlabeled learning. Advances in Neural Information Processing Systems. 2020;33:2903–13.

[75] Titouan V, Courty N, Tavenard R, Flamary R. International Conference on Machine Learning. 6275–84.

[76] Flamary R, et al. Pot: Python optimal transport. Journal of Machine Learning Research. 2021;22:1–8.

[77] The UniProt, C. UniProt: the universal protein knowledgebase. Nucleic acids research.2017;45, D158–D169. doi: 10.1093/nar/gkw1099 PMC5210571 27899622

[78] Eisenberg D, Weiss RM, Terwilliger TC, Wilcox W. Faraday Symposia of the Chemical Society. Royal Society of Chemistry. 109–20.

[79] Bordin N, Sillitoe I, Nallapareddy V, Rauer C, Lam SD, Waman VP, et al. AlphaFold2 reveals commonalities and novelties in protein structure space for 21 model organisms. Commun Biol. 2023;6(1):160. doi: 10.1038/s42003-023-04488-9 36755055 PMC9908985

[80] Heinig M, Frishman D. STRIDE: A web server for secondary structure assignment from known atomic coordinates of proteins. Nucleic Acids Res. 2004;32(Web Server issue):W500-2. doi: 10.1093/nar/gkh429 15215436 PMC441567

[81] Cingolani P, Platts A, Wang LL, Coon M, Nguyen T, Wang L, et al. A program for annotating and predicting the effects of single nucleotide polymorphisms, SnpEff: SNPs in the genome of Drosophila melanogaster strain w1118; iso-2; iso-3. Fly (Austin). 2012;6(2):80–92. doi: 10.4161/fly.19695 22728672 PMC3679285

[82] Sherry ST, Ward MH, Kholodov M, Baker J, Phan L, Smigielski EM, et al. dbSNP: The NCBI database of genetic variation. Nucleic Acids Res. 2001;29(1):308–11. doi: 10.1093/nar/29.1.308 11125122 PMC29783

