# Supplementary figures and images for: Exploring the structural lexicon of the Proteome via Metric Geometry
Source: PLoS Comput Biol. 2026 Jun 30;22(6):e1014487. doi: 10.1371/journal.pcbi.1014487 (PMC13336474; doi:10.1371/journal.pcbi.1014487)

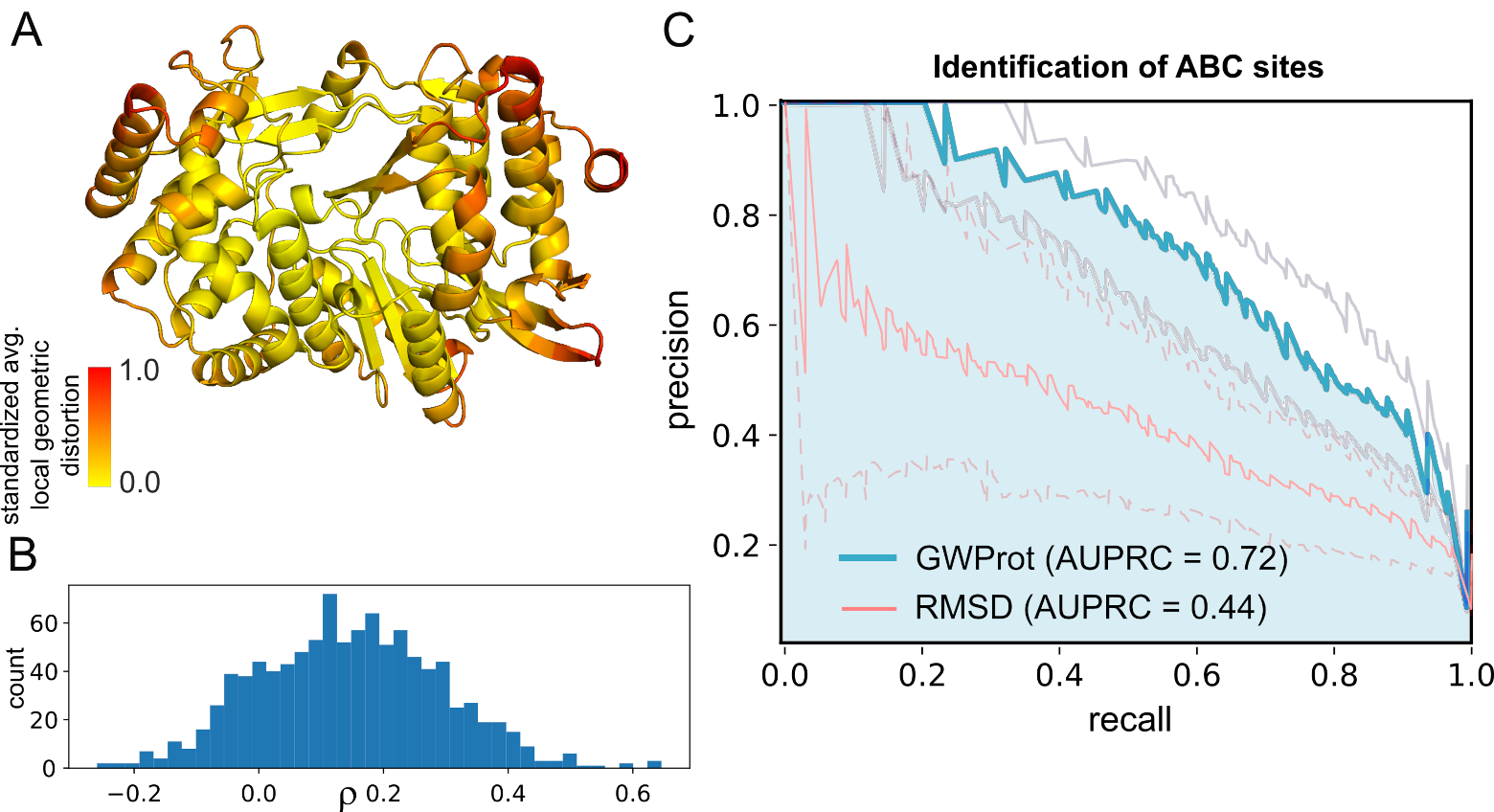

Supplement: S1 Fig — A) Example of an RdRp core domain from Hepacivirus hominis (GenPept ID AFD18577) colored by average GW local geometric distortion. Regions of low distortion, mostly located at the catalytic core, are structurally conserved. B) Distribution of Spearman correlation coefficients between local geometric distortion and residue-level deviations from RMSD-minimizing rigid-body alignment for each RdRp core domain in each pairwise alignment. C) Median, 20%, and 80% percentile precision-recall curves for predicting A, B, or C sites based on average GW local geometric distortion (blue) and average residue-level deviations from RMSD-minimizing rigid-body alignment (red) across 97 randomly selected RdRps. AUPRC: area under the precision-recall curve; RMSD: root-mean-square deviation. (TIFF) [file pcbi.1014487.s001.tiff]

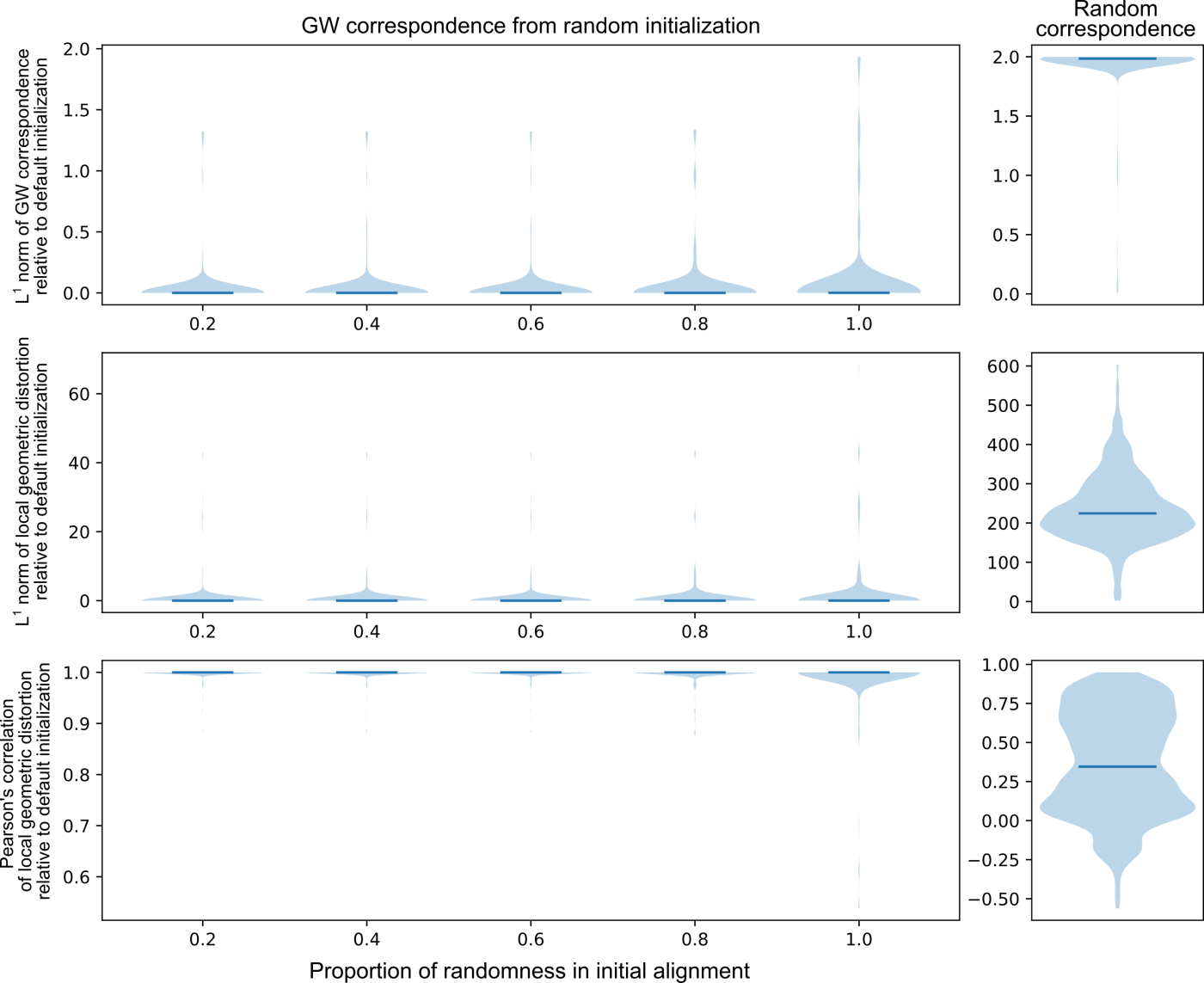

Supplement: S2 Fig — For each of 50 randomly selected pairs of RdRps, we compared the GW correspondence and local geometric distortions obtained from the default initialization P0 with those obtained from initializations of the form aPr+(1−a)P0, where a∈[0,1] denotes the proportion of randomness and Pr is a random initial alignment generated by uniform sampling from the space of feasible correspondences using hit-and-run Markov chain Monte Carlo. For reference, the GW correspondences were also compared with random correspondences. (TIFF) [file pcbi.1014487.s002.tiff]

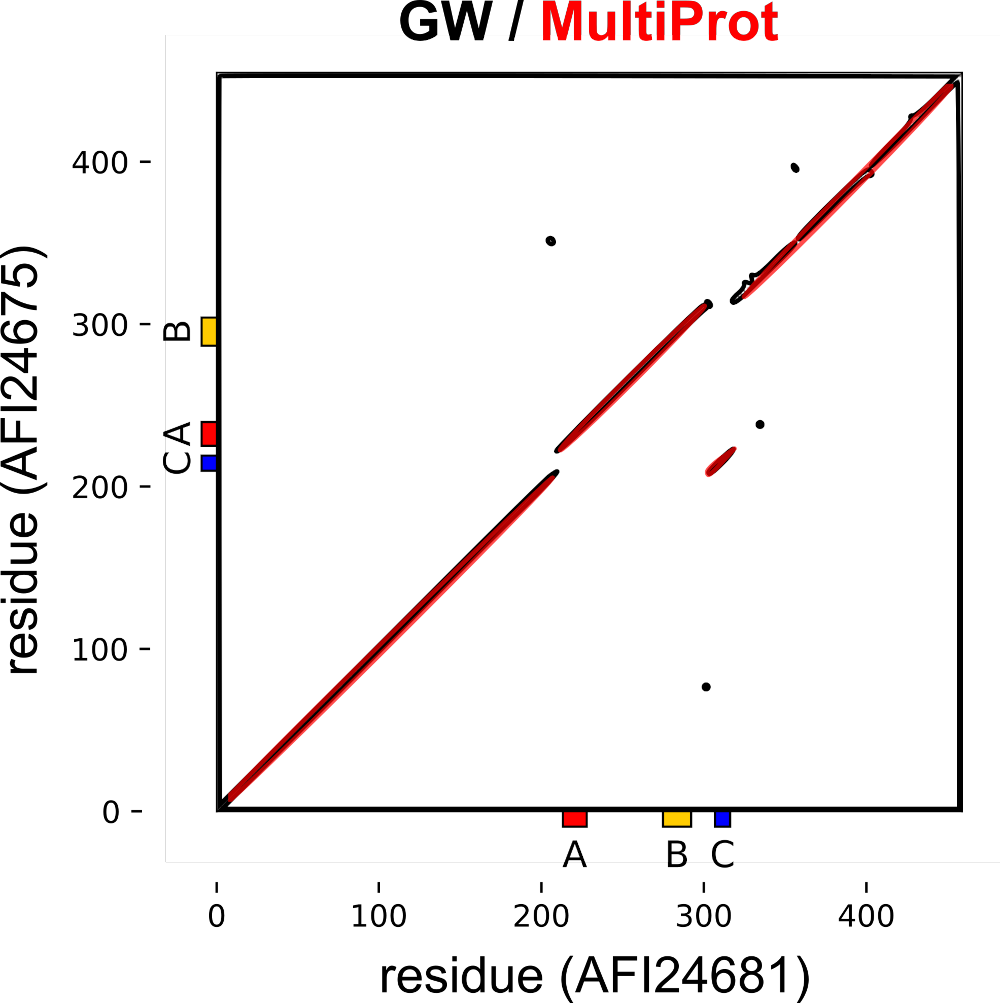

Supplement: S3 Fig — (TIFF) [file pcbi.1014487.s003.tiff]

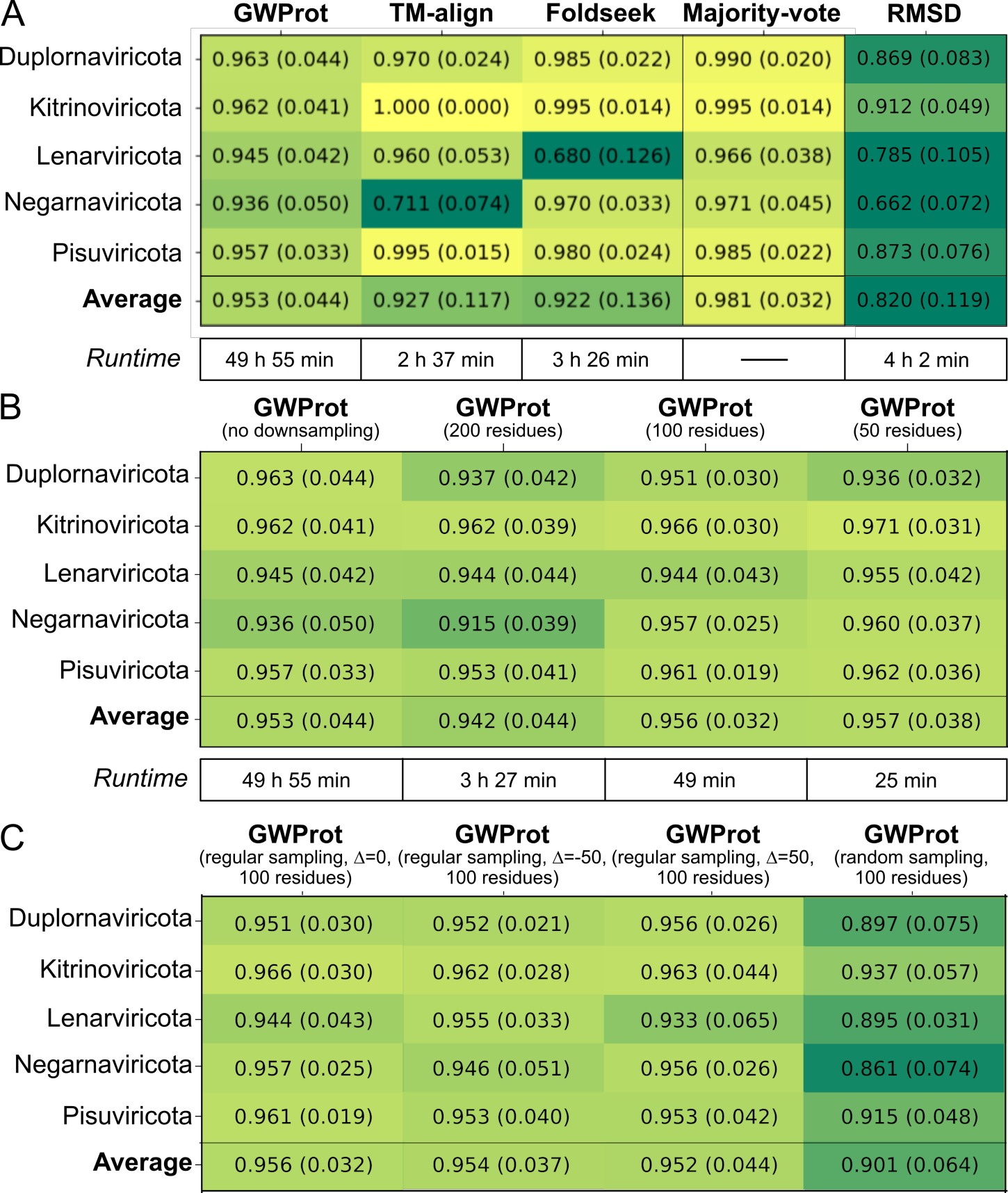

Supplement: S4 Fig — A) Shown are the average 10-fold cross-validation MCC values and standard deviations (in parenthesis) for a k = 3 nearest neighbor classifier trained on structural embedding spaces produced by GWProt, TM-align, Foldseek, and rigid-body alignment minimizing RMSD. The classifiers were evaluated on the task of distinguishing RdRp core domain structures belonging to phyla absent from the training data from non-RdRp decoys with high amino acid sequence similarity to bona fide RdRp core domains. The MCC of a majority-vote classifier combining GWProt, TM-align, and Foldseek is also shown. Runtimes on a standard 8-core desktop computer using parallelization are also indicated. B) Average 10-fold cross-validation MCC values and standard deviations are shown for GWProt without downsampling and with regular downsampling to 200, 100, and 50 residues. Runtimes on a standard 8-core desktop computer using parallelization are also indicated. C) Average 10-fold cross-validation MCC values and standard deviations are shown for GWProt applied to 100 regularly downsampled residues with different shifts (Δ=−50%, 0%, +50% of the distance between sampled residues) and to 100 randomly sampled residues with equal probability. (TIFF) [file pcbi.1014487.s004.tiff]

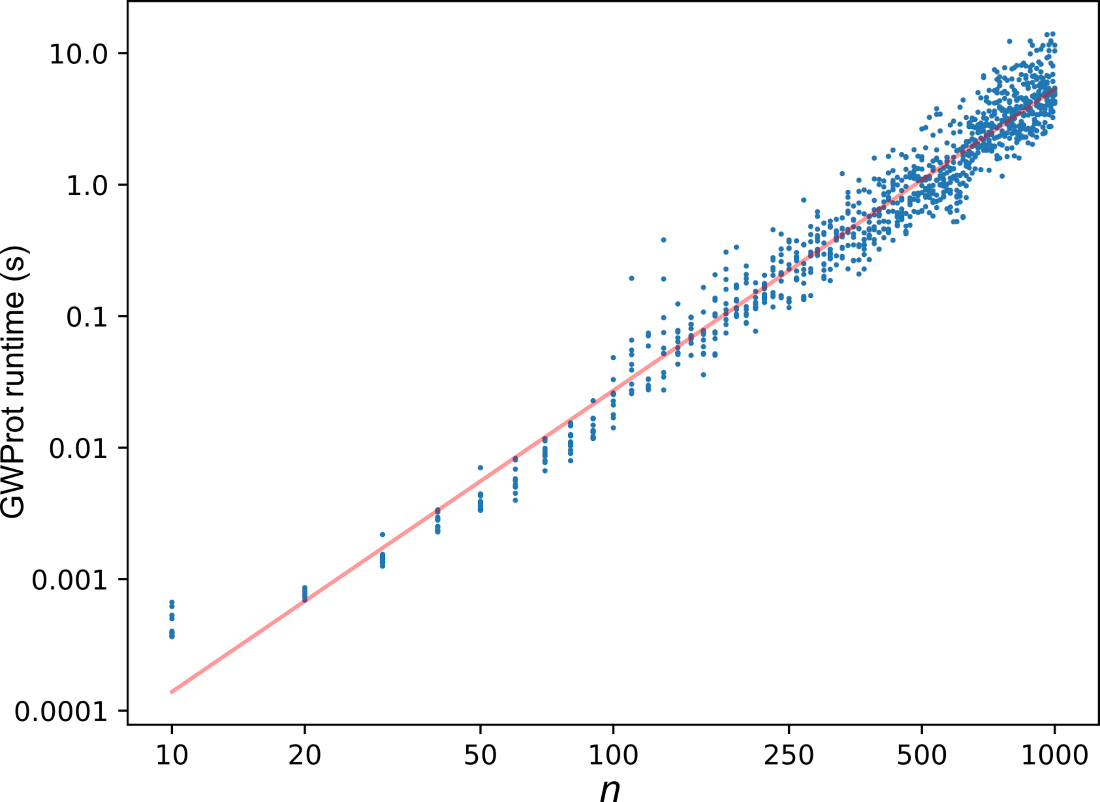

Supplement: S5 Fig — Runtime of GWProt, in seconds, as a function of the number of residues for 1,000 randomly sampled RdRp polypeptide-chain pairs. The fitted line follows the empirical scaling law t≃7×10^−7^ n2.3. (TIFF) [file pcbi.1014487.s005.tiff]

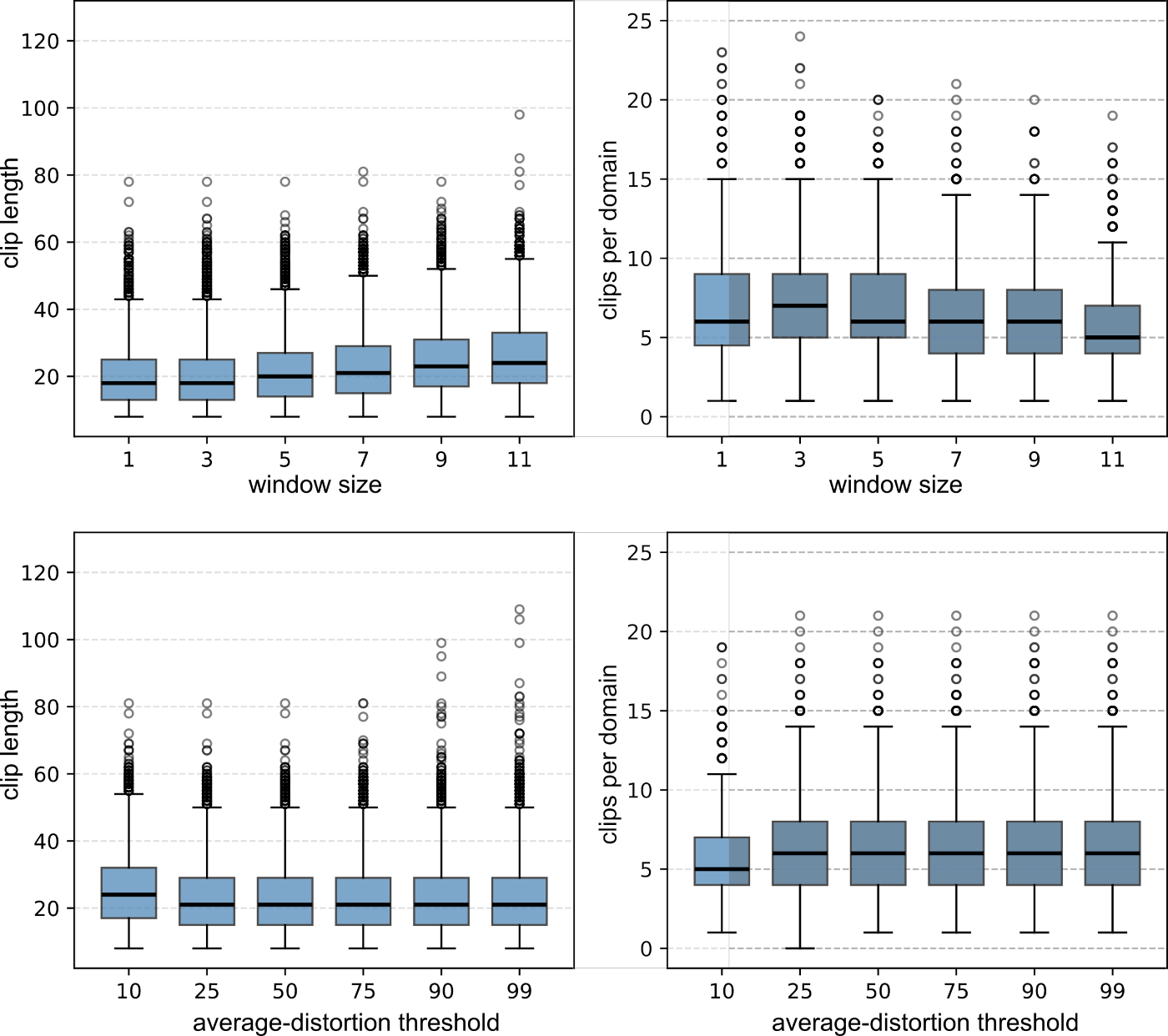

Supplement: S6 Fig — The algorithm was applied to 50 randomly selected homologous superfamilies (1,667 domains total) across varying rolling-window sizes (keeping the threshold on average geometric distortion fixed at 50%) and thresholds on average local geometric distortion (keeping the window size fixed at 7 residues). (TIFF) [file pcbi.1014487.s006.tiff]

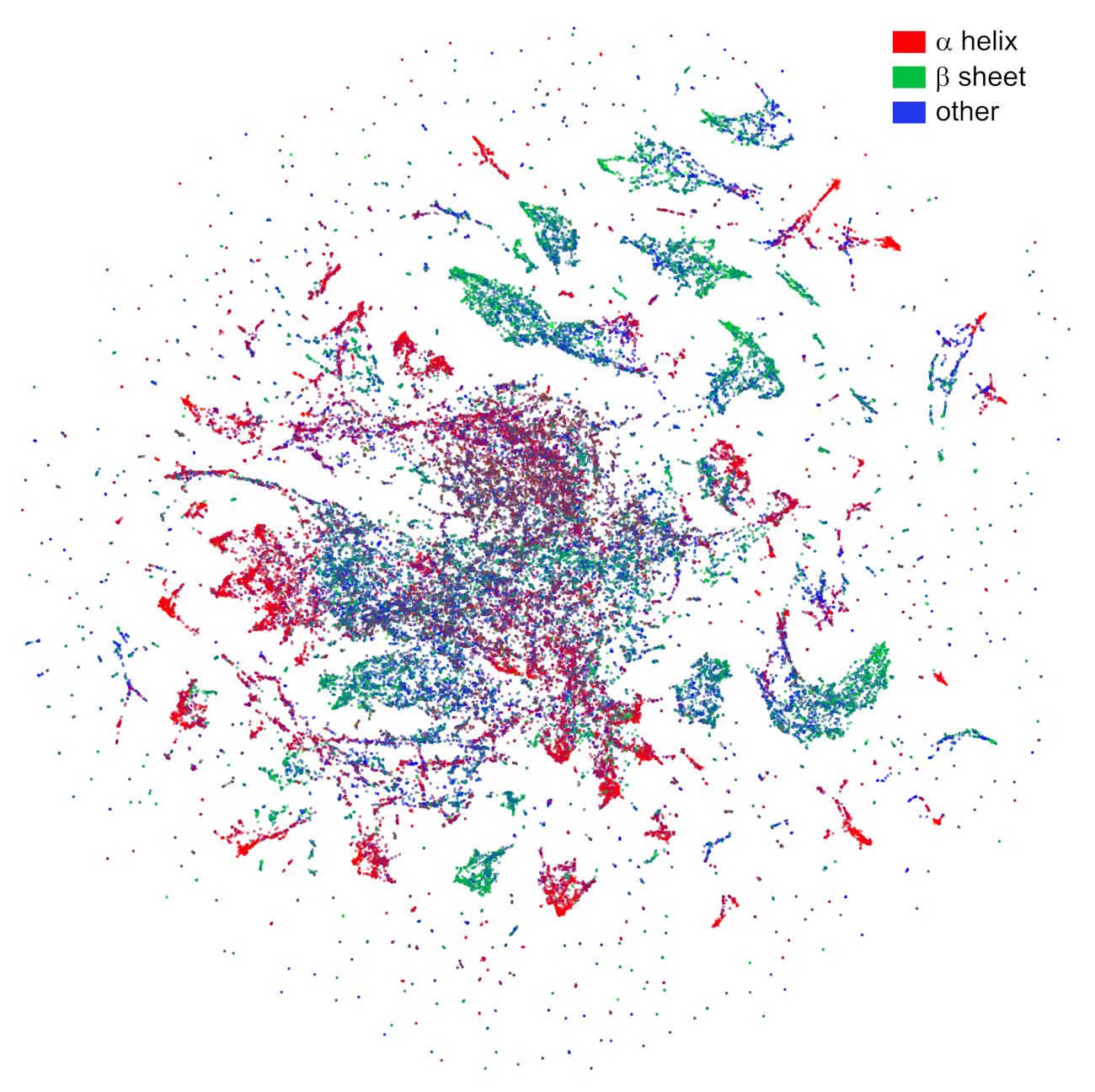

Supplement: S7 Fig — (TIFF) [file pcbi.1014487.s007.tiff]

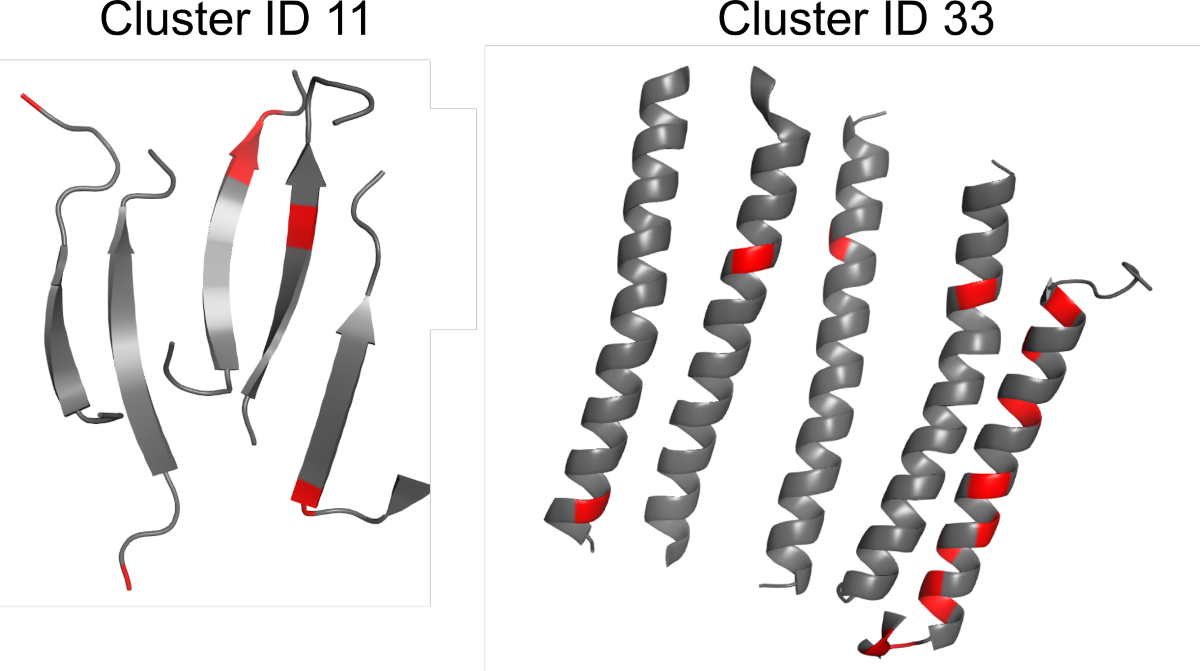

Supplement: S8 Fig — Positions with known pathogenic or likely pathogenic SNVs are indicated in red. (TIFF) [file pcbi.1014487.s008.tiff]
