# Supplementary material for: Exploring the structural lexicon of the Proteome via Metric Geometry
Source: PLoS Comput Biol. 2026 Jun 30;22(6):e1014487. doi: 10.1371/journal.pcbi.1014487 (PMC13336474; doi:10.1371/journal.pcbi.1014487)
Supplement: Attachment — Submitted filename: Reviewer_Comments.pdf [file pcbi.1014487.s014.pdf]

Response to the Reviewers' Comments

Comments from Reviewer #1:

The authors introduce the methods/algorithms from the area of metric geometry to the study of protein structures. Problems involving protein structure comparisons can be formulated within a metric geometry framework (could probably be used for RNA/DNA and chemicals, too). Apparently some of the algorithms necessary to efficiently solve such problems from this realm were developed not too long ago, and the authors demonstrate the utility of these methods with a number of very interesting applications to computational structural biology.

Their applications include identifying the switch regions in an oncoprotein, functional domains in evolutionarily distant viral proteins, permuted motifs in a group of homologous proteins, recognizing RNA polymerases from different viral phyla, and looking for conserved small structural motifs across the human proteome.

A very nice feature of this approach is that the method does not rely on sequence order, so that structure similarity between motifs that may be sequence-wise permuted in different structures can still be recognized. They give a nice example of this with the RdRps (RNA polymerases). The paper is very well written and the results appear to be reasonable. My comments are mainly concerned with adding a bit more of explanation to clarify a few things.

We thank the reviewer for the constructive comments and positive feedback. In the revised manuscript, we have provided additional clarifications to improve the presentation of the methods and analyses. The specific changes are detailed below:

The introductory paragraph in Methods could use some clarification. The matrix T is supposed to be a 0/1-valued matrix and it is stated that each row sum should be equal to 1, as well as each column sum. But this requirement on the row and column sums implies that T must be a square matrix, i.e. m equal to n. I think that should be made clear at this point in the paper.

We thank the reviewer for pointing this out. In the original version of the manuscript, there was a typo in the constraints that the coupling matrix T must satisfy. The correct constraints are $\sum_i T_{i,k} = 1/m$ and $\sum_k T_{i,k} = 1/n$, where n and m denote the number of residues in the two proteins being compared. Thus, T is not required to be square when $n \neq m$. In addition, the entries of T are not binary, but take values in the interval [0,1], so that T can be interpreted as a coupling (that is, a probability distribution) with uniform marginals rather than as a 0/1 assignment matrix.

We have corrected the typo and added a clarifying sentence in the revised manuscript.

This implies that one must be careful about choosing the subsets of atoms to compare. I think this is the simple reason for some of the limitations mentioned at the end of the Discussion.

We agree with the reviewer that, in practice, care is required in choosing which atoms or residues to compare. Although the couplings matrices need not be square and, in principle, all atoms or residues could be included, computational feasibility requires restricting the analysis to a subset. In our analyses, we focused on alpha-carbon atoms, which provide a representation of the protein backbone, and in some cases further restricted the comparison to a subset of residues. We

therefore agree that it is important to assess the extent to which the results depend on the choice of downsampling strategy.

In the revised manuscript, we have evaluated the relationship between downsampling, runtime, and accuracy in the viral phyla identification analysis presented in the subsection "*GW distances support the discovery of previously unseen viral phyla*". The results are shown in Supplementary Figure 4C of the revised manuscript (see also Reviewer #3, Comment 6). We find that, in this setting, the results are robust to substantial downsampling, while providing a major improvement in runtime. In particular, with 50 regularly sampled residues, GWProt is faster than both TM-Align (fast option) and Foldseek. We speculate that this robustness arises because the RdRp core domain contains sufficient large-scale structural information to support phylum-level identification. Consistent with this interpretation, the results are also robust to offsets in the sampled residues. By contrast, accuracy is substantially reduced when uniform random sampling is used instead of regular sampling, as reflected in the MCC scores and their standard deviation:

B

	**GWProt** (no downsampling)	**GWProt** (200 residues)	**GWProt** (100 residues)	**GWProt** (50 residues)
Duplornaviricota	0.963 (0.044)	0.937 (0.042)	0.951 (0.030)	0.936 (0.032)
Kitrinoviricota	0.962 (0.041)	0.962 (0.039)	0.966 (0.030)	0.971 (0.031)
Lenarviricota	0.945 (0.042)	0.944 (0.044)	0.944 (0.043)	0.955 (0.042)
Negarnaviricota	0.936 (0.050)	0.915 (0.039)	0.957 (0.025)	0.960 (0.037)
Pisuviricota	0.957 (0.033)	0.953 (0.041)	0.961 (0.019)	0.962 (0.036)
**Average**	0.953 (0.044)	0.942 (0.044)	0.956 (0.032)	0.957 (0.038)
Runtime	49 h 55 min	3 h 27 min	49 min	25 min

C

	**GWProt** (regular sampling, δ=0, 100 residues)	**GWProt** (regular sampling, δ=-50, 100 residues)	**GWProt** (regular sampling, δ=50, 100 residues)	**GWProt** (random sampling, 100 residues)
Duplornaviricota	0.951 (0.030)	0.952 (0.021)	0.956 (0.026)	0.897 (0.075)
Kitrinoviricota	0.966 (0.030)	0.962 (0.028)	0.963 (0.044)	0.937 (0.057)
Lenarviricota	0.944 (0.043)	0.955 (0.033)	0.933 (0.065)	0.895 (0.031)
Negarnaviricota	0.957 (0.025)	0.946 (0.051)	0.956 (0.026)	0.861 (0.074)
Pisuviricota	0.961 (0.019)	0.953 (0.040)	0.953 (0.042)	0.915 (0.048)
**Average**	0.956 (0.032)	0.954 (0.037)	0.952 (0.044)	0.901 (0.064)

We have revised subsection "*GW distances support the discovery of previously unseen viral phyla*" to incorporate these results.

For the problem of computing the matrix to optimize the GW cost, are there any computational complexity results for the approximation algorithms? I.e. how good are the approximations and what about the running times? Whatever is known would be good to mention; perhaps this could inspire improved algorithms to be developed.

We solve the non-convex optimization problems in Eqs. (2) and (3) using the conditional-gradient (Frank-Wolfe) approach described by Vayer et al.[1] and implemented in the POT[2] and CAJAL[3]

libraries. In this approach, each iteration requires computing the gradient of the GW objective and solving a classical optimal transport subproblem. For the squared-loss formulation, the gradient computation scales as $\mathcal{O}(mn^2 + m^2n)$, and $n \simeq m$ the OT subproblem is commonly taken to have roughly cubic worst-case scaling (often quoted as $\mathcal{O}(n^3 \log n)$ for network flow-based solvers like the one used in our work). Because the GW objective is non-convex, these algorithms do not in general provide a global approximation guarantee; rather, they are guaranteed to converge to a stationary point of the optimization problem.

To assess runtime empirically in the setting relevant to protein structure alignment, we randomly sampled pairs of RdRps and decoys from the dataset used in the subsection *"GW distances enhance the discovery of previously unseen viral phyla"*. From these, we selected polypeptide chains of length n, ranging from 10 to 1,000 residues, and ran GWProt in a single CPU thread to align 1,000 polypeptide chain pairs in total. In this benchmark, runtime in seconds was well fit by by $t \simeq 7 \times 10^{-7}\, n^{2.3}$, substantially better than a naïve cubic worst-case expectation. In these analyses, the conditional-gradient stopping tolerance on the relative error was set to 10^{-9}.

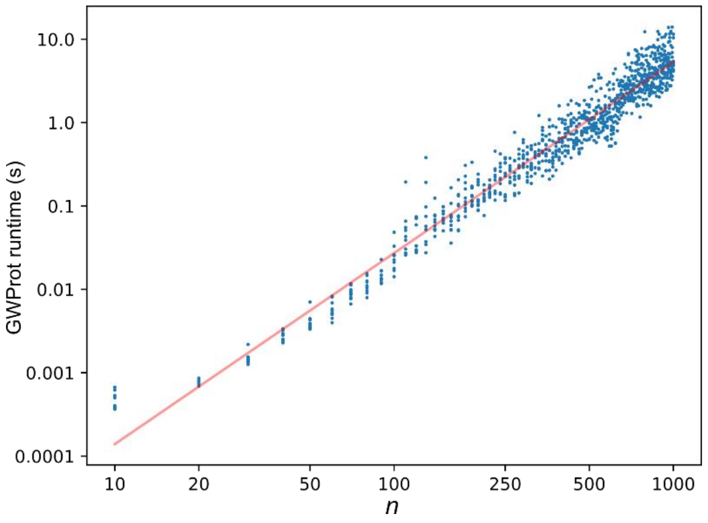

In the revised manuscript, we now explicitly discuss these computational considerations in subsection *"GWProt: Gromov-Wasserstein correspondences for protein structural alignment"* and include the empirical runtime analyses in the new Supplementary Figure 5.

On p. 19 they refer to 'Duplonaviricota', I think this is a little typo and should probably be 'Duplornaviricota'.

We thank the reviewer for pointing this out. We have corrected this typo in the revised manuscript.

For the RdRps test, it says on p. 20 that they "… augmented this dataset with 300 non-RdRp decoy structures that clustered at 97% amino acid sequence identity …" Do they mean the decoys by themselves formed a cluster at pairwise 97% sequence identity?

The decoys were drawn from a curated structural benchmark of viral and non-viral palm-domain structures assembled in previous work by Drs. Robert Edgar and Artem Babaian[4,5] to benchmark algorithms for discriminating viral RdRps from their closest non-viral homologs, primarily Group II introns. This benchmark includes SCOP-annotated palm-domain PDB structures together with

selected high-quality AlphaFold models (based on pLDDT score) of GenBank proteins identified as close homologs by BLAST- or HMM-based searches, including both canonical and permuted palm-domain architectures. The dataset was then redundancy-reduced by sequence-homology clustering.

After consulting with the authors of that benchmark dataset, we learned that a variable sequence-homology threshold was used for clustering in this dataset. Thus, the statement in the original manuscript should not be interpreted as meaning that the decoys themselves formed a cluster at 97% pairwise amino acid sequence identity. Our confusion arose because the earlier description in Babaian et al.[4] used a 97% threshold in the initial version of the approach.

In the revised manuscript, we have corrected this point and clarified the description of the benchmark dataset accordingly.

I am not an expert on viruses, but given that the results in Fig 2B look quite good, is it in fact true that the RdRps are a basis for the classification of these riboviral phyla to begin with? I think that is fine but think that it would be good to state this, to clarify for other non-experts.

We thank the reviewer for this suggestion. The RdRp is indeed a hallmark gene of RNA viruses and a major basis for the higher-level classification of riboviral phyla, although additional genomic and biological features are also taken into account. We therefore agree that stating this explicitly would help clarify the significance of the results in Figure 2B for readers. In this regard, we believe that the analysis presented in Supplementary Figure 4, where we evaluate the identification of RdRps from previously unseen riboviral phyla, is more representative of a practical discovery setting. We have revised subsection *"Local geometric distortion identifies functional domains in evolutionarily distant viral proteins"* accordingly.

Comments from Reviewer #2:

This paper introduces GWProt, a computational framework that applies Gromov-Wasserstein (GW) and fused Gromov-Wasserstein (FGW) optimal transport to protein structure alignment. The authors propose a local geometric distortion measure derived from the GW objective to identify structurally conserved or variable regions. The approach is demonstrated on several biological applications, including detection of conformational switch regions in KRAS, identification of motifs in RdRps, and discovery of recurrent short structural fragments across human protein domains. While the approach is interesting, I have several concerns on the lack of algorithmic details, computational efficiency, stability of the GW/FGW outcomes, and the genuine benefit of solving such a computationally expensive problem over simple and cheap baselines.

We thank the reviewer for the constructive comments and suggestions. In the revised manuscript, we have clarified the algorithmic details, expanded the analyses of computational efficiency and stability, and added quantitative comparisons with simple baselines, as detailed below.

Specific points:

1. The feasibility definition of T is problematic. When n does not equal m, it is impossible to enforce \sum_i T_{i,k}=\sum_k T_{i,k}=1. Depending on how the feasibility of T is defined, Eq. (3) may not

be equivalent to the more commonly used formulation where the Wasserstein part is given by <\delta, T>.

We thank the reviewer for pointing this out. As noted above in response to Reviewer #1, the constraints on the coupling matrix T were stated incorrectly in the original version of the manuscript. The correct marginal constraints are $\sum_i T_{i,k} = 1/m$ and $\sum_k T_{i,k} = 1/n$, where n and m are the number of residues in the two proteins being compared. We have corrected this in the revised manuscript. With these corrected constraints, T belongs to the standard set of admissible couplings with uniform marginals, and Eq. (3) is equivalent to the standard FGW formulation of Vayer et al.[6].

2. Related to the previous point, if the feasible set is indeed the standard feasible set used in balanced OT with uniform initial and terminal distributions, how would one interpret the soft matching represented by T? For example, when n and m are significantly different (say n > m), balanced OT will force splitting mass of points in Y to X, causing large distortion values even if Y is identical to a subgraph of X. Could the authors discuss or explore the usage of unbalanced or partial OT setup in this application?

We agree that the inability of standard GW/FGW to represent partial structural alignments between proteins of substantially different sizes is an important limitation, as we now state more clearly in the last paragraph of the Discussion. In the balanced setting, the transport plan must satisfy fixed marginal constraints, so when one protein contains an approximately matching substructure embedded within a much larger structure, the coupling is forced to distribute mass beyond the homologous region.

Unbalanced and partial GW[7-9] (UGW and PGW) are natural extensions that could in principle alleviate this limitation. However, in the implementations we tested, we found that they are not yet sufficiently robust or efficient to be consistently useful for protein structure alignment at scale. In particular, parameter choices were often highly sensitive to the specific protein pair being aligned, the non-convex optimization landscape made initialization specifically difficult when the proteins differed substantially in size (the identity-based initialization used in GWProt often fails to guide the optimizer toward the correct partial match in the unbalanced or partial setting, converging to a poor local minimum), and runtime was often much higher than for standard GW/FGW. For these reasons, we did not include unbalanced or partial GW in this manuscript. Instead, we focused on setting in which the aligned structures have comparable sizes, including previously annotated regions with similar lengths (RdRp core domains and CATH domains) and allosteric changes within the same protein (the KRAS example).

To assess these issues more directly, we compared balanced GW with UGW (implemented in the POT library) using the Src kinase family as a test system. Specifically, we considered 3 isolated SH2 domain structures (Src SH2, 103 amino acids, PDB ID: 1SHB; Lck SH2, 105 amino acids, PDB ID: 1LKK; and Grb2 SH2, 100 amino acids, PDB ID: 3K2M) together with the full-length Src kinase (449 amino acids; PDB ID: 2SRC), which contains SH2, SH3 and kinase domains. When comparing isolated SH2 domains to one another, both GW and UGW recovered the expected near-identity correspondences, although UGW was approximately 5x slower than GW:

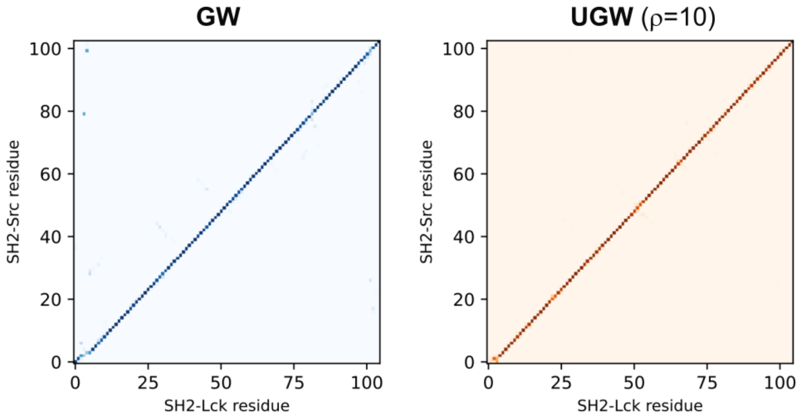

When comparing isolated SH2 domains to the full-length Src, balanced GW, as expected, distributed mass broadly across the larger structure rather than restricting it to the homologous SH2 domain. UGW sometimes concentrated above-chance mass on the correct SH2 domain for particular values of the KL-divergence parameter ρ, but this behavior was not stable across proteins. For example, for $\rho = 10$, 48% of the Src-full mass was concentrated on the SH2 domain (compared to 22% under a uniform coupling) when aligning Grb2 SH2 to full-length Src, but only 1% was concentrated on the SH2 domain when aligning Src SH2 to full-length Src. Furthermore, even in cases when mass was concentrated on the correct domain, the within-domain residue correspondence remained weak:

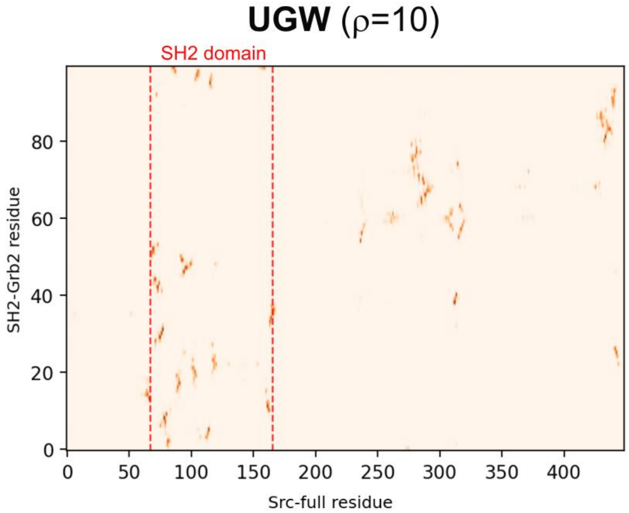

We also tested PGW, which explicitly controls the fraction of transported mass m. In our hands, however, only the entropic implementation converged, the dependence on m was erratic, the SH2 domain captured only about 10% of the transported mass, and runtime was two to three orders of magnitude slower than GW.

Together, these results suggest that, although unbalanced and partial GW are promising directions for extending GW alignment beyond full matching, current implementations or formulations are not yet sufficiently robust or efficient for protein structure alignment.

We have revised the last paragraph of the Discussion to clarify this point.

3. The algorithm used is unclear. Network simplex is for linear problems and GW and FGW are quadratic. Solving GW or FGW typically involves an outer loop with inner steps to iteratively solve linear problems. The full algorithm including the linearization, should be described.

We solve the GW and FGW optimization problems using the Frank-Wolfe (conditional gradient) method described in Vayer et al.[1] and implemented in the POT[2] and CAJAL[3] libraries. In this approach, the quadratic objective is iteratively linearized around the current coupling, and each resulting linear optimal transport subproblem is then solved using the network simplex algorithm. We now include a description of this full optimization procedure in the Methods section of the revised manuscript under the subheading "*Protein structural alignment with Gromov-Wasserstein correspondences*".

4. The decomposition in Eqs. (4) and (5) are immediate algebraic restructuring of the loss term. I believe that this is rarely used in practice because GW and FGW are non-convex. Typical algorithms guarantee convergence of objective value to a local minimum but the coupling matrix is often only guaranteed to converge to a stationary point. Thus GW and FGW are more trustworthy as a global metric and for representing the global coupling structure. Zooming into distortions due to individual points could be highly unstable. Could the authors clarify this point, or at least demonstrate numerically that the residue level interpretation is stable when different initializations of GW/FGW algorithms are used?

We thank the reviewer for this comment. Because GW and FGW are non-convex, the conditional-gradient solver used in our work is, in general, guaranteed to converge to a stationary point rather than to a global optimum. As a result, different initializations can in principle lead to different coupling matrices, especially when the compared structures exhibit exact or approximate symmetries.

We therefore agree with the reviewer that the residue-level interpretation should be supported by a numerical stability analysis. To assess this, we randomly selected 50 pairs of RdRp core domains from the dataset used in subsection "*Local geometric distortion identifies functional domains in evolutionarily distant viral proteins*". For each pair, we compared the coupling matrix obtained from the default near-identity initialization P_0 with the coupling matrices obtained from initializations of the form $aP_r + (1-a)P_0$, where $a \in [0,1]$ and P_r is a random feasible coupling generated by uniform sampling hit-and-run Markov chain Monte Carlo. We also compared these solutions to random feasible couplings.

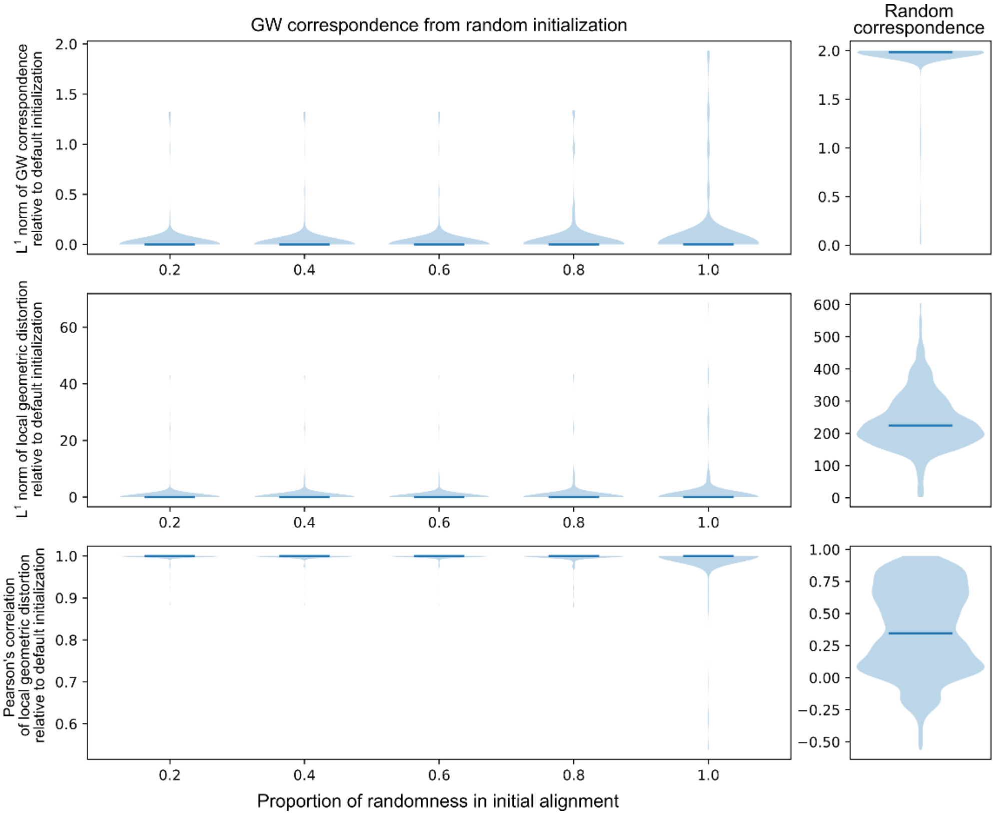

The results indicate a high degree of empirical stability in both the local geometric distortions and the coupling matrices across different initializations, with Pearson correlation coefficients typically exceeding 0.99 and identical coupling matrices obtained in most cases. We have included this analysis in Supplementary Figure 2 of the revised manuscript.

We also note that, because local geometric distortion is nonnegative, the L^1-difference between the residue-level distortion profiles associated with two couplings is bounded by the sum of their GW objective values, up to the normalization convention used in the definition of the local distortion:

$$\sum_i \left|D(x_i) - \widetilde{D}(x_i)\right| \leq \sum_i \left(D(x_i) + \widetilde{D}(x_i)\right) = 4\left(GW^2 + \widetilde{GW}^2\right)$$

Thus, when two alignments both achieve very small GW objective values, their residue-level distortion profiles must also be close. Although this observation is generally weaker than the empirical stability analysis above, it is consistent with the high stability observed in practice for strongly homologous structures.

5. Computing GW/FGW is often computationally costly, even with subsampling, when used to compute pairwise distances. Could the authors clarify the computational complexity and empirical computing time to conduct an analysis at such scale? The ~2000 proteins implies several million of FGW computations and the clips clustering analysis involves pairwise FGW computation for over 20000 samples. Could the authors clarify if any efficient approximation was used?

In all analyses presented in the manuscript, we solved the standard GW/FGW optimization problem using the Frank-Wolfe (conditional-gradient) method implemented in the Python Optimal Transport (POT)[2] and CAJAL[3] libraries, rather than using low-rank or quantized GW approximations. The main practical strategy we used to reduce runtime was downsampling of the protein structures, not approximation of the GW/FGW solver itself.

In this optimization framework, each outer conditional-gradient iteration requires solving a linear OT subproblem. In the case in which both structures have approximately the same number of residues, this OT subproblem has $\mathcal{O}(n^3 \log n)$ worst-case scaling on the number of residues n, although in practice the network simplex algorithm often converges substantially faster than this asymptotic bound suggests[10]. In the revised manuscript, we now include an empirical evaluation of GW runtimes in the context of protein structure alignment (Supplementary Fig. 5; see also Reviewer #1, Comment 4), showing that runtime scales approximately as $n^{2.3}$ in this setting.

During the course of this project, we also explored existing approximation strategies, including low-rank[11] and quantized[12] GW. However, for the effective sizes of the aligned structures considered here, we found that downsampling was generally more effective, substantially reducing runtime while having only a limited effect on alignment accuracy (see Reviewer #3, Comment 6). In the subsection *“Local geometric distortion identifies functional domains in evolutionarily distant viral proteins”*, we aligned only the RdRp core domains rather than full proteins; these domains have an average size of 169 residues. Similarly, in the subsection *“Local geometric distortions across the human proteome reveal the structural lexicon underlying functional domains”*, the clips have an average length of 22 residues. For point clouds of this size, low-rank and quantized approximations did not provide a practical runtime advantage, and individual GW/FGW computations were fast. The total wall-clock time therefore reflected the very large number of pairwise comparisons (3.8 million for the 2,777 RdRps core domains and 2.2 billion for the 66,937 clips), rather than a high cost for each individual alignment. On a standard 8-core desktop computer, the full analyses took approximately 1 day for the 2,777 RdRp core domains downsampled to 200 residues and approximately 1 week for the 66,937 clips.

To address this point, we have added clarifying text to the revised manuscript, including the new empirical runtime and downsampling analysis in Supplementary Figure 5 and additional clarification in subsection “*Local geometric distortions across the human proteome reveal the structural lexicon underlying functional domains*”.

6. It is unclear how the many hyperparameters in the study were selected. For example, the \alpha in FGW, number of downsampling, 7-residue window, median distortion as a threshold, etc. A clarification of all the parameter choices and some sensitivity analysis study of the parameters would be helpful.

The main hyperparameters in our analyses are the FGW weighting parameter α, the number of sampled residues, and, in analyses based on local geometric distortion, the threshold used to define low-distortion regions. We agree that clarifying how these parameters were chosen is important, and we have added a new subsection entitled *“Hyperparameter selection”* to the Methods section of the revised manuscript.

To select α, we examined the relative magnitudes of the feature-matching and GW terms in the FGW objective across our datasets, and found that the GW term was typically one order of magnitude larger than the feature-matching term. This identified a practical range of α values for which the two terms contribute on comparable scales. Based on this analysis, we chose $\alpha = 0.2$, and visually inspected a small random subset of alignments as a sanity check to confirm that the resulting correspondences were reasonable. We then evaluated sensitivity to this choice and found that the results were relatively stable across the range of α values. For example, in the analysis of RdRp ABC motif prediction shown in Figure 2D of the manuscript, the area under the precision-recall curve remained largely stable over the range of $\alpha \in [0.05, 0.5]$:

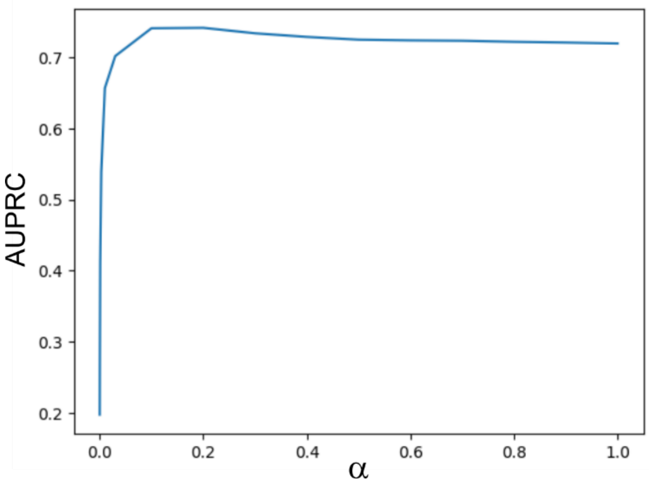

The number of sampled residues was chosen based on computational constraints, using the largest value that could be run on a standard desktop computer within a reasonable time. We have also evaluated the sensitivity to this choice and found that the results are relatively stable with respect to the degree of downsampling, as shown in in Supplementary Figure 4B of the revised manuscript.

Analyses based on local geometric distortion also require choosing a threshold to define structurally conserved regions. This threshold controls the tradeoff between type I and type II errors. In KRAS switch-region analysis and RdRp ABC-domain analysis presented in Figures 1C and 2D of the manuscript, we varied this threshold to construct precision-recall curves. In the analysis of structural motifs across human functional domains, however, such threshold sweeps were not computationally feasible at scale. In that setting, we used the median distortion as a conservative default threshold.

Finally, for analyses involving a 7-residue window, this choice was introduced as a local smoothing scale to reduce sensitivity to residue-level noise while preserving short segments. We verified that the results are relatively stable for nearby window sizes, as described in our response to the next point.

7. Related to the previous point, the clips are chosen by rolling a 7-residue window and thresholding the average distortion of the window by the median distortion of the domain. This median threshold is rather arbitrary. I suggest exploring some more rigorous statistical strategy.

We thank the reviewer for this suggestion. We have now clarified the rationale for parameter choices in the new subsection *"Hyperparameter selection"* of the Methods section. In the revised manuscript, we have also evaluated the robustness of the results to different parameter choices by rerunning the clipping algorithm on 50 randomly selected homologous superfamilies comprising 1,667 domains in total. Specifically, we varied both the rolling-window size and the average-distortion threshold, and found that the distributions of clip length and of the number of clips per domain remained relatively stable across a broad range of parameter values:

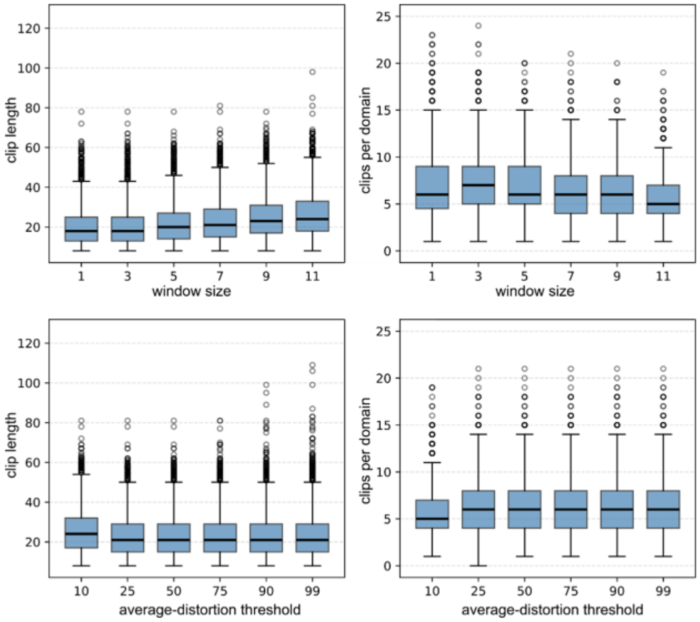

These results are presented in the new Supplementary Figure 6 of the revised manuscript.

8. In the only example with comparison to two existing methods (TM-align and Foldseek), GWProt is ranked the 3rd in 3 out of 5 and the 2nd in 2 out of 5 tasks. So the improvement over existing approaches is rather insignificant. I wonder how much benefit does solving original GW/FGW problem really bring. It would be interesting to see if a much simpler approach would achieve similar performance. For example, optimize a rigid-body motion and let I={(i_k,j_k)} be pairs of nearest neighbor points from the two proteins after performing the rigid-body motion on protein B, and minimize \sum_k \delta(i_k,j_k). A coupling T, if needed, can be recovered by just solving a Wasserstein OT using the optimal rotation+translation.

We agree with the reviewer that, in this benchmark, GW-based distances perform competitively with TM-align and Foldseek rather than uniformly outperforming them. The main conclusion of this analysis is therefore not that GW-based distances dominate existing methods across the board, but that they capture complementary information that improves performance when combined with other approaches, as supported by the strong performance of the majority-vote classifier. We have revised this section of the manuscript to better reflect that conclusion.

At the same time, solving the GW/FGW problem provides a clear advantage over much simpler baselines. To assess this directly, we compared GWProt, TM-align, and Foldseek against a rigid-

body alignment baseline that minimizes RMSD, as suggested by the reviewer. The results show that GWProt substantially outperforms this baseline, as reflected by markedly higher MCC values:

	**GWProt**	**TM-align**	**Foldseek**	Majority-vote	Rigid alignmnt
Duplornaviricota	0.963 (0.044)	0.970 (0.024)	0.985 (0.022)	0.990 (0.020)	0.869 (0.083)
Kitrinoviricota	0.962 (0.041)	1.000 (0.000)	0.995 (0.014)	0.995 (0.014)	0.912 (0.049)
Lenarviricota	0.945 (0.042)	0.960 (0.053)	0.680 (0.126)	0.966 (0.038)	0.785 (0.105)
Negarnaviricota	0.936 (0.050)	0.711 (0.074)	0.970 (0.033)	0.971 (0.045)	0.662 (0.072)
Pisuviricota	0.957 (0.033)	0.995 (0.015)	0.980 (0.024)	0.985 (0.022)	0.873 (0.076)
**Average**	0.953 (0.044)	0.927 (0.117)	0.922 (0.136)	0.981 (0.032)	0.820 (0.119)

We have included these new results in Supplementary Figure 4A of the revised manuscript.

Comments from Reviewer #3:

The work reported in this manuscript relies on extremely large all-vs-all GW computations. However, there is no report of the computational cost as a reference point for comparison, or for independent replication – ie. hardware resources used, runtimes. This would shed light as to how deployable the GW computations are across a wider variety of computing environments. This aspect is particularly important because GW is known to be computationally expensive and the authors do mention this fact. Therefore, I believe the authors should provide this context and reference to allow for readers to assess replicability or deployability in their own computing environments.

We thank the reviewer for this suggestion. In the revised manuscript, we now report the runtimes of main large-scale analyses, together with the hardware used, to provide context for reproducibility and deployability. Specifically, these computations were performed on a standard desktop computer equipped with an 8-core 3.70 GHz Intel Xeon CPU using parallelization.

The analyses in our manuscript focus on relatively small protein structures. In the subsection "*Local geometric distortion identifies functional domains in evolutionarily distant viral proteins*", the RdRp core domains have an average length of 503 residues and were downsampled to 200 residues for large-scale pairwise analysis. In the subsection *"Local geometric distortions across the human proteome reveal the structural lexicon underlying functional domains",* the all-vs-all comparison was performed on 66,937 clips, which have an average length of 22 residues. For structures of this size, individual GW/FGW computations are fast. Accordingly, the total wall-clock time primarily reflects the very large number of pairwise comparisons rather than the cost of any single alignment: approximately 1 day for the 2,777 RdRps core domains (3.8 million pairwise comparisons) and 1 week for the 66,937 clips (2.2 billion pairwise comparisons).

In addition, we now include an empirical analysis of runtime as a function of the number of residues (see Reviewer #1, Comment 4), showing that the runtime in seconds for each pairwise alignment scales approximately as $n^{2.3}$, where n is the number of residues. This analysis is shown in the new Supplementary Figure 5 of the revised manuscript.

The section "GW distances enhance the discovery of previously unseen viral phyla" is potentially interesting, but the conclusions should be softened and be aligned to the fact that the reported performance differences between GWProt, TM-align, and Foldseek are relatively small. Furthermore, the authors explicitly acknowledge that GWProt is not the best performer in any individual phylum.

We agree that the original text overstated the magnitude of the performance differences between GWProt, TM-align, and Foldseek. In response, we have revised this section to soften the conclusions and better align them with the data. Specifically, we now emphasize that GW-based distances perform competitively with TM-align and Foldseek, and that their main advantage in this analysis is that they provide complementary information that improves performance when combined with these methods (see all Reviewer #2, Comment 8). This interpretation is supported by the strong performance of the majority-vote classifier, which outperformed each individual method.

Although the embedding construction differs substantially between methods, no attempt was made to harmonize post-processing (e.g. distance normalization, embedding method).It is therefore unclear whether the observed differences are due to the alignment method itself, or the way the structural space is constructed. I recommend either providing a more careful normalization and calibration across methods, or reframing this section as a complementarity demonstration rather than a performance comparison. The majority-vote classifier result should be emphasized more.

We agree with the reviewer that each method produces a different structural embedding space because of the distinct nature of the underlying algorithms. This was, in fact, our rationale for using a k-nearest-neighbor classifier for the performance evaluation, as it depends only on the relative ordering of pairwise distances and is therefore insensitive to normalization or embedding choices, provided these do not alter that ordering. In the revised manuscript, we now make this point explicit and, as suggested by the reviewer, have revised this section to emphasize the complementarity contribution of GWProt and the utility of the majority-vote classifier.

The structural motif analysis is interesting, but remains largely descriptive. Beyond the two example motif clusters there is no direct demonstration that newly identified motifs correspond to known binding sites, known functional sites / functional microenvironments, or known fold-specific motifs. Given the existing literature on local structural motifs and microenvironments, a short comparative analysis (for example against existing motif libraries or known structural signatures) would substantially strengthen the biological interpretation. For example, have they considered looking at variations or conservations of 3D motifs/substructures, such as those that can be found by tools like Rasmot 3D Pro, ProBis, Pyscomotif, within the same clips or whether different clips can contain conserved 3D motifs?

We thank the reviewer for this suggestion. We agree that a comparison against existing structural motif libraries would strengthen the biological interpretation, and we have made our best effort to address this point. However, the 3D motifs represented in existing libraries are generally larger in scale than the clips considered here and are often formed by multiple non-contiguous regions of the protein rather than by a short contiguous segment. For example, the serine protease catalytic

site motif (M-CSA entry 173) highlighted in the PyScoMotif tutorial is a 3-residue His-Asp-Ser motif whose residues are typically distributed over approximately 150 residues. By contrast, the clips in our analysis have an average length of 22 residues and correspond to short recurrent structural fragments, more akin to "letters" than to full 3D functional motifs or "words".

To address this point in the revised manuscript, we have performed a comparative analysis using annotations from the Mechanism and Catalytic Site Atlas (M-CSA). Rather than attempting to match full 3D catalytic motifs, we have focused on residues with known catalytic roles. This analysis shows that clips are significantly enriched for residues with catalytic roles relative to non-clipped regions of the CATH domains (odds = 1.12, Fisher's exact test p-value = 10^{-34}). In addition, in some of the clusters identified in our analysis, residues with known catalytic roles appear to play similar functional roles within the active site according to the M-CSA annotations. In the revised manuscript, we highlight one such example: a cluster of 11-residue clips in which all residues with known catalytic roles participate in transition-state stabilization through hydrogen bonding.

We have also clarified in the revised manuscript the conceptual relationship between the clips studied here and related structural-motif concepts from the previous literature.

The physical interpretation of local geometric distortion could be further clarified because readers might wonder how strongly it correlates with local RMSD or local distance-matrix deviations.

Local geometric distortion differs conceptually from local RMSD in several important respects. In particular, local geometric distortion is defined in terms of intra-protein distances and is naturally compatible with soft (probabilistic) correspondences, whereas local RMSD is defined in terms of between-protein distances and requires a hard (deterministic) correspondence between residues. In the special case where two proteins X and Y have the same length n and the transport plan T is a scaled permutation matrix σ/n (that is, each residue is mapped entirely to a single residue), the resulting local geometric distortion is proportional to the squared difference in intra-protein distances:

$$\mathrm{D}(\mathrm{x_i}) = \mathrm{D}(\mathrm{y}_{\sigma(\mathrm{i})}) = \frac{1}{n^2}\sum_{\mathrm{j}}\left(\mathrm{d_X}(\mathrm{x_i},\mathrm{x_j}) - \mathrm{d_Y}(\mathrm{y}_{\sigma(\mathrm{i})},\mathrm{y}_{\sigma(\mathrm{j})})\right)^2$$

Thus, local geometric distortion measures how well the local relational geometry around a residue is preserved under the alignment, rather than how close two residues are in Euclidean space after rigid superposition.

To compare local geometric distortion and local RMSD quantitatively, we considered the 97 RdRp core domains from subsection "*Local geometric distortion identifies functional domains in evolutionarily distant viral proteins*" and, for each protein in each pairwise alignment, computed the Spearman correlation across residues between local geometric distortion and per-residue deviation after rigid alignment. Although the mean Spearman correlation was positive, it was small for most proteins (mean $\rho = 0.15$), indicating that the two quantities are only weakly related:

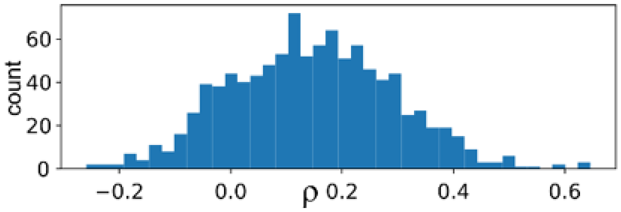

This is consistent with our new comparison between GWProt to rigid-body alignment methods minimizing RMSD in the identification of previously unseen riboviral phyla (see Reviewer #2, Comment 8), in which RMSD-based alignment performed substantially worse than GWProt, TM-align, and Foldseek. Likewise, replacing local geometric distortion with local RMSD for identification of the A, B, and C sites within the RdRp core domain reduced the AUPRC from 0.72 to 0.44:

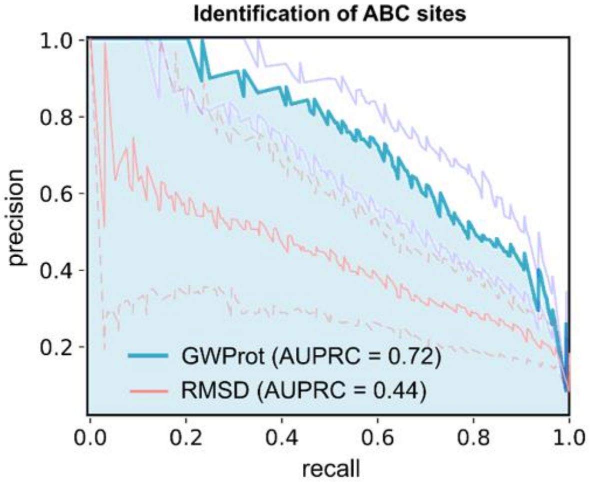

In the revised manuscript, we have clarified the distinction between local geometric distortion and local RMSD. We have also included the Spearman correlation analysis in Supplementary Figure 1B and the comparison of precision-recall curves for local geometric distortion and RMSD in Supplementary Figure 1C.

I accept that downsampling of RdRps to 200 residues is necessary for computational reasons, but the manuscript does not assess how much information is lost as a result of the downsampling. This could be a useful reference point for readers.

In the revised manuscript, we now explicitly assess the tradeoff between downsampling, runtime, and predictive accuracy in the viral phyla identification analysis presented in subsection "*GW distances support the discovery of previously unseen viral phyla*". As shown in Supplementary Figure 4B, downsampling can substantially reduce runtime while preserving most of the predictive accuracy over a broad range of sampling levels, as reflected in the average MCC scores and their standard deviations (see also Reviewer #1, Comment 3):

B

	GWProt (no downsampling)	GWProt (200 residues)	GWProt (100 residues)	GWProt (50 residues)
Duplornaviricota	0.963 (0.044)	0.937 (0.042)	0.951 (0.030)	0.936 (0.032)
Kitrinoviricota	0.962 (0.041)	0.962 (0.039)	0.966 (0.030)	0.971 (0.031)
Lenarviricota	0.945 (0.042)	0.944 (0.044)	0.944 (0.043)	0.955 (0.042)
Negarnaviricota	0.936 (0.050)	0.915 (0.039)	0.957 (0.025)	0.960 (0.037)
Pisuviricota	0.957 (0.033)	0.953 (0.041)	0.961 (0.019)	0.962 (0.036)
**Average**	0.953 (0.044)	0.942 (0.044)	0.956 (0.032)	0.957 (0.038)
Runtime	49 h 55 min	3 h 27 min	49 min	25 min

In addition, we have assessed the sensitivity of the results to the choice of downsampling strategy and found that regular sampling yields more accurate and more stable results than uniform random sampling (Supplementary Figure 4C).

The term "clip" is intuitive but non-standard in structural bioinformatics. A short comparison with "fragments", "structural motifs", "local motifs", or "tertiary/3D substructures" used in previous literature would improve clarity.

We thank the reviewer for this suggestion. In the revised manuscript, we have added a short discussion placing the clips used in our study in the context of related terms from the previous literature. Similar to traditional local motifs[13-15], clips occupy a conceptual space between classical fixed-length backbone fragments[16-18] and full functional domains as classified by CATH[19] and SCOP[20], thereby bridging a level of structural organization between secondary structure elements and tertiary folds. However, unlike traditional local motifs, which are typically defined by geometric recurrence across structurally diverse proteins, clips are delineated through pairwise geometric comparison within homologous superfamilies using GW correspondences, and therefore capture regions that have been structurally preserved across evolutionarily related domains. Thus, clusters of structurally homologous clips, which we refer to in the manuscript as "structural motifs", parallel the notion of recurrent local structural elements from previous works[14,21], while being derived from evolutionarily conserved sub-domain units identified through alignment-derived geometric distortions.

References

1 Titouan, V., Courty, N., Tavenard, R. & Flamary, R. in *International Conference on Machine Learning*. 6275-6284 (PMLR).

2 Flamary, R. *et al.* Pot: Python optimal transport. *Journal of Machine Learning Research* **22**, 1-8 (2021).

3 Govek, K. W. *et al.* CAJAL enables analysis and integration of single-cell morphological data using metric geometry. *Nature communications* **14**, 3672, doi:10.1038/s41467-023-39424-2 (2023).

4 Babaian, A. & Edgar, R. Ribovirus classification by a polymerase barcode sequence. *PeerJ* **10**, e14055, doi:10.7717/peerj.14055 (2022).

5 Edgar, R. C. *et al.* Petabase-scale sequence alignment catalyses viral discovery. *Nature* **602**, 142-147, doi:10.1038/s41586-021-04332-2 (2022).

6 Vayer, T., Chapel, L., Flamary, R., Tavenard, R. & Courty, N. Fused Gromov-Wasserstein Distance for Structured Objects. *Algorithms* **13**, 212 (2020).

7 Séjourné, T., Vialard, F.-X. & Peyré, G. The unbalanced gromov wasserstein distance: Conic formulation and relaxation. *Advances in Neural Information Processing Systems* **34**, 8766-8779 (2021).

8 Thual, A. *et al.* Aligning individual brains with fused unbalanced Gromov Wasserstein. *Advances in neural information processing systems* **35**, 21792-21804 (2022).

9 Chapel, L., Alaya, M. Z. & Gasso, G. Partial optimal tranport with applications on positive-unlabeled learning. *Advances in Neural Information Processing Systems* **33**, 2903-2913 (2020).

10 Dong, Y., Gao, Y., Peng, R., Razenshteyn, I. & Sawlani, S. A study of performance of optimal transport. *arXiv preprint arXiv:2005.01182* (2020).

11 Scetbon, M., Peyré, G. & Cuturi, M. in *International Conference on Machine Learning*. 19347-19365 (PMLR).

12 Chowdhury, S., Miller, D. & Needham, T. 811-827 (Springer International Publishing).

13 de Brevern, A. G., Valadie, H., Hazout, S. & Etchebest, C. Extension of a local backbone description using a structural alphabet: a new approach to the sequence-structure relationship. *Protein Sci* **11**, 2871-2886, doi:10.1110/ps.0220502 (2002).

14 Mackenzie, C. O., Zhou, J. & Grigoryan, G. Tertiary alphabet for the observable protein structural universe. *Proceedings of the National Academy of Sciences of the United States of America* **113**, E7438-E7447, doi:10.1073/pnas.1607178113 (2016).

15 Nepomnyachiy, S., Ben-Tal, N. & Kolodny, R. Global view of the protein universe. *Proceedings of the National Academy of Sciences of the United States of America* **111**, 11691-11696, doi:10.1073/pnas.1403395111 (2014).

16 Kolodny, R., Koehl, P., Guibas, L. & Levitt, M. Small libraries of protein fragments model native protein structures accurately. *J Mol Biol* **323**, 297-307, doi:10.1016/s0022-2836(02)00942-7 (2002).

17 Simons, K. T., Kooperberg, C., Huang, E. & Baker, D. Assembly of protein tertiary structures from fragments with similar local sequences using simulated annealing

and Bayesian scoring functions. *J Mol Biol* **268**, 209-225, doi:10.1006/jmbi.1997.0959 (1997).

18 Gront, D., Kulp, D. W., Vernon, R. M., Strauss, C. E. & Baker, D. Generalized fragment picking in Rosetta: design, protocols and applications. *PLoS One* **6**, e23294, doi:10.1371/journal.pone.0023294 (2011).

19 Orengo, C. A. *et al.* CATH--a hierarchic classification of protein domain structures. *Structure* **5**, 1093-1108, doi:10.1016/s0969-2126(97)00260-8 (1997).

20 Murzin, A. G., Brenner, S. E., Hubbard, T. & Chothia, C. SCOP: a structural classification of proteins database for the investigation of sequences and structures. *J Mol Biol* **247**, 536-540, doi:10.1006/jmbi.1995.0159 (1995).

21 Alva, V., Soding, J. & Lupas, A. N. A vocabulary of ancient peptides at the origin of folded proteins. *Elife* **4**, e09410, doi:10.7554/eLife.09410 (2015).
