# Supplementary material for: Exploring the structural lexicon of the Proteome via Metric Geometry
Source: PLoS Comput Biol. 2026 Jun 30;22(6):e1014487. doi: 10.1371/journal.pcbi.1014487 (PMC13336474; doi:10.1371/journal.pcbi.1014487)
Supplement: Peer review history

# Peer review: Decision Letter 0

**DOI:** 10.1371/journal.pcbi.1014487.r001

**Authors:** Jeffrey Skolnick, Ferhat Ay

5 Mar 2026

PCOMPBIOL-D-25-02291

Exploring the Structural Lexicon of the Proteome via Metric Geometry

PLOS Computational Biology

Dear Dr. Gonzalez Camara,

Thank you for submitting your manuscript to PLOS Computational Biology. After careful consideration, we feel that it has merit but does not fully meet PLOS Computational Biology's publication criteria as it currently stands. Therefore, we invite you to submit a revised version of the manuscript that addresses the points raised during the review process.

Please submit your revised manuscript by May 05 2026 11:59PM. If you will need more time than this to complete your revisions, please reply to this message or contact the journal office at ploscompbiol@plos.org. When you're ready to submit your revision, log on to https://www.editorialmanager.com/pcompbiol/ and select the 'Submissions Needing Revision' folder to locate your manuscript file.

Please include the following items when submitting your revised manuscript:

* A letter that responds to each point raised by the editor and reviewer(s). You should upload this letter as a separate file labeled 'Response to Reviewers'. This file does not need to include responses to formatting updates and technical items listed in the 'Journal Requirements' section below.

* A marked-up copy of your manuscript that highlights changes made to the original version. You should upload this as a separate file labeled 'Revised Manuscript with Track Changes'.

* An unmarked version of your revised paper without tracked changes. You should upload this as a separate file labeled 'Manuscript'.

If you would like to make changes to your financial disclosure, competing interests statement, or data availability statement, please make these updates within the submission form at the time of resubmission. Guidelines for resubmitting your figure files are available below the reviewer comments at the end of this letter

We look forward to receiving your revised manuscript.

Kind regards,

Jeffrey Skolnick

Academic Editor

PLOS Computational Biology

Ferhat Ay

Section Editor

PLOS Computational Biology

**Journal Requirements:**

1) We ask that a manuscript source file is provided at Revision. Please upload your manuscript file as a .doc, .docx, .rtf or .tex. If you are providing a .tex file, please upload it under the item type u2018LaTeX Source Fileu2019 and leave your .pdf version as the item type u2018Manuscriptu2019.

2) Please provide an Author Summary. This should appear in your manuscript between the Abstract (if applicable) and the Introduction, and should be 150-200 words long. The aim should be to make your findings accessible to a wide audience that includes both scientists and non-scientists. Sample summaries can be found on our website under Submission Guidelines:

https://journals.plos.org/ploscompbiol/s/submission-guidelines#loc-parts-of-a-submission

3) Please upload all main figures as separate Figure files in .tif or .eps format. For more information about how to convert and format your figure files please see our guidelines:

https://journals.plos.org/ploscompbiol/s/figures

4) We have noticed that you have uploaded Supporting Information files, but you have not included a list of legends. Please add a full list of legends for your Supporting Information files after the references list.

5) Please amend your detailed Financial Disclosure statement. This is published with the article. It must therefore be completed in full sentences and contain the exact wording you wish to be published.

- State the initials, alongside each funding source, of each author to receive each grant. For example: "This work was supported by the National Institutes of Health (####### to AM; ###### to CJ) and the National Science Foundation (###### to AM)."

- State what role the funders took in the study. If the funders had no role in your study, please state: "The funders had no role in study design, data collection and analysis, decision to publish, or preparation of the manuscript.".

If you did not receive any funding for this study, please simply state: u201cThe authors received no specific funding for this work.u201d

**Reviewers' comments:**

Reviewer's Responses to Questions

**Comments to the Authors:**

**Please note here if the review is uploaded as an attachment.**

Reviewer #1: The authors introduce the methods/algorithms from the area of metric geometry to the study of protein structures. Problems involving protein structure comparisons can be formulated within a metric geometry framework (could probably be used for RNA/DNA and chemicals, too). Apparently some of the algorithms necessary to efficiently solve such problems from this realm were developed not too long ago, and the authors demonstrate the utility of these methods with a number of very interesting applications to computational structural biology.

Their applications include identifying the switch regions in an oncoprotein, functional domains in evolutionarily distant viral proteins, permuted motifs in a group of homologous proteins, recognizing RNA polymerases from different viral phyla, and looking for conserved small structural motifs across the human proteome.

A very nice feature of this approach is that the method does not rely on sequence order, so that structure similarity between motifs that may be sequence-wise permuted in different structures can still be recognized. They give a nice example of this with the RdRps (RNA polymerases).

The paper is very well written and the results appear to be reasonable. My comments are mainly concerned with adding a bit more of explanation to clarify a few things.

The introductory paragraph in Methods could use some clarification. The matrix T is supposed to be a 0/1-valued matrix and it is stated that each row sum should be equal to 1, as well as each column sum. But this requirement on the row and column sums implies that T must be a square matrix, i.e. m equal to n. I think that should be made clear at this point in the paper. This implies that one must be careful about choosing the subsets of atoms to compare. I think this is the simple reason for some of the limitations mentioned at the end of the Discussion.

For the problem of computing the matrix to optimize the GW cost, are there any computational complexity results for the approximation algorithms? I.e. how good are the approximations and what about the running times? Whatever is known would be good to mention; perhaps this could inspire improved algorithms to be developed.

On p. 19 they refer to ‘Duplonaviricota’, I think this is a little typo and should probably be ‘Duplornaviricota’.

For the RdRps test, it says on p. 20 that they “… augmented this dataset with 300 non-RdRp decoy structures that clustered at 97% amino acid sequence identity …” Do they mean the decoys by themselves formed a cluster at pairwise 97% sequence identity?

I am not an expert on viruses, but given that the results in Fig 2B look quite good, is it in fact true that the RdRps are a basis for the classification of these riboviral phyla to begin with? I think that is fine but think that it would be good to state this, to clarify for other non-experts.

Reviewer #2: This paper introduces GWProt, a computational framework that applies Gromov-Wasserstein (GW) and fused Gromov-Wasserstein (FGW) optimal transport to protein structure alignment. The authors propose a local geometric distortion measure derived from the GW objective to identify structurally conserved or variable regions. The approach is demonstrated on several biological applications, including detection of conformational switch regions in KRAS, identification of motifs in RdRps, and discovery of recurrent short structural fragments across human protein domains. While the approach is interesting, I have several concerns on the lack of algorithmic details, computational efficiency, stability of the GW/FGW outcomes, and the genuine benefit of solving such a computationally expensive problem over simple and cheap baselines.

Specific points:

1. The feasibility definition of T is problematic. When n does not equal m, it is impossible to enforce \sum_i T_{i,k}=\sum_k T_{i,k}=1. Depending on how the feasibility of T is defined, Eq. (3) may not be equivalent to the more commonly used formulation where the Wasserstein part is given by <\delta, T>.

2. Related to the previous point, if the feasible set is indeed the standard feasible set used in balanced OT with uniform initial and terminal distributions, how would one interpret the soft matching represented by T? For example, when n and m are significantly different (say n > m), balanced OT will force splitting mass of points in Y to X, causing large distortion values even if Y is identical to a subgraph of X. Could the authors discuss or explore the usage of unbalanced or partial OT setup in this application?

3. The algorithm used is unclear. Network simplex is for linear problems and GW and FGW are quadratic. Solving GW or FGW typically involves an outer loop with inner steps to iteratively solve linear problems. The full algorithm including the linearization, should be described.

4. The decomposition in Eqs. (4) and (5) are immediate algebraic restructuring of the loss term. I believe that this is rarely used in practice because GW and FGW are non-convex. Typical algorithms guarantee convergence of objective value to a local minimum but the coupling matrix is often only guaranteed to converge to a stationary point. Thus GW and FGW are more trustworthy as a global metric and for representing the global coupling structure. Zooming into distortions due to individual points could be highly unstable. Could the authors clarify this point, or at least demonstrate numerically that the residue level interpretation is stable when different initializations of GW/FGW algorithms are used?

5. Computing GW/FGW is often computationally costly, even with subsampling, when used to compute pairwise distances. Could the authors clarify the computational complexity and empirical computing time to conduct an analysis at such scale? The ~2000 proteins implies several million of FGW computations and the clips clustering analysis involves pairwise FGW computation for over 20000 samples. Could the authors clarify if any efficient approximation was used?

6. It is unclear how the many hyperparameters in the study were selected. For example, the \alpha in FGW, number of downsampling, 7-residue window, median distortion as a threshold, etc. A clarification of all the parameter choices and some sensitivity analysis study of the parameters would be helpful.

7. Related to the previous point, the clips are chosen by rolling a 7-residue window and thresholding the average distortion of the window by the median distortion of the domain. This median threshold is rather arbitrary. I suggest exploring some more rigorous statistical strategy.

8. In the only example with comparison to two existing methods (TM-align and Foldseek), GWProt is ranked the 3rd in 3 out of 5 and the 2nd in 2 out of 5 tasks. So the improvement over existing approaches is rather insignificant. I wonder how much benefit does solving original GW/FGW problem really bring. It would be interesting to see if a much simpler approach would achieve similar performance. For example, optimize a rigid-body motion and let I={(i_k,j_k)} be pairs of nearest neighbor points from the two proteins after performing the rigid-body motion on protein B, and minimize \sum_k \delta(i_k,j_k). A coupling T, if needed, can be recovered by just solving a Wasserstein OT using the optimal rotation+translation.

Reviewer #3: The work reported in this manuscript relies on extremely large all-vs-all GW computations. However, there is no report of the computational cost as a reference point for comparison, or for independent replication – ie. hardware resources used, runtimes. This would shed light as to how deployable the GW computations are across a wider variety of computing environments. This aspect is particularly important because GW is known to be computationally expensive and the authors do mention this fact. Therefore, I believe the authors should provide this context and reference to allow for readers to assess replicability or deployability in their own computing environments.

The section “GW distances enhance the discovery of previously unseen viral phyla” is potentially interesting, but the conclusions should be softened and be aligned to the fact that the reported performance differences between GWProt, TM-align, and Foldseek are relatively small. Furthermore, the authors explicitly acknowledge that GWProt is not the best performer in any individual phylum.

Although the embedding construction differs substantially between methods, no attempt was made to harmonize post-processing (e.g. distance normalization, embedding method).It is therefore unclear whether the observed differences are due to the alignment method itself, or the way the structural space is constructed. I recommend either providing a more careful normalization and calibration across methods, or reframing this section as a complementarity demonstration rather than a performance comparison. The majority-vote classifier result should be emphasized more.

The structural motif analysis is interesting, but remains largely descriptive. Beyond the two example motif clusters there is no direct demonstration that newly identified motifs correspond to known binding sites, known functional sites / functional microenvironments, or known fold-specific motifs. Given the existing literature on local structural motifs and microenvironments, a short comparative analysis (for example against existing motif libraries or known structural signatures) would substantially strengthen the biological interpretation. For example, have they considered looking at variations or conservations of 3D motifs/substructures, such as those that can be found by tools like Rasmot 3D Pro, ProBis, Pyscomotif, within the same clips or whether different clips can contain conserved 3D motifs?

The physical interpretation of local geometric distortion could be further clarified because readers might wonder how strongly it correlates with local RMSD or local distance-matrix deviations.

I accept that downsampling of RdRps to 200 residues is necessary for computational reasons, but the manuscript does not assess how much information is lost as a result of the downsampling. This could be a useful reference point for readers.

The term “clip” is intuitive but non-standard in structural bioinformatics. A short comparison with “fragments”, “structural motifs”, “local motifs”, or “tertiary/3D substructures” used in previous literature would improve clarity.

**********

**Have the authors made all data and (if applicable) computational code underlying the findings in their manuscript fully available?**

The PLOS Data policy requires authors to make all data and code underlying the findings described in their manuscript fully available without restriction, with rare exception (please refer to the Data Availability Statement in the manuscript PDF file). The data and code should be provided as part of the manuscript or its supporting information, or deposited to a public repository. For example, in addition to summary statistics, the data points behind means, medians and variance measures should be available. If there are restrictions on publicly sharing data or code —e.g. participant privacy or use of data from a third party—those must be specified.

Reviewer #1: Yes

Reviewer #2: Yes

Reviewer #3: Yes

**********

PLOS authors have the option to publish the peer review history of their article (what does this mean?). If published, this will include your full peer review and any attached files.

If you choose “no”, your identity will remain anonymous but your review may still be made public.

**Do you want your identity to be public for this peer review?**  For information about this choice, including consent withdrawal, please see our Privacy Policy.

Reviewer #1: No

Reviewer #2: No

Reviewer #3: No

[NOTE: If reviewer comments were submitted as an attachment file, they will be attached to this email and accessible via the submission site. Please log into your account, locate the manuscript record, and check for the action link "View Attachments". If this link does not appear, there are no attachment files.]

**Figure resubmission:**

While revising your submission, we strongly recommend that you use PLOS’s NAAS tool (https://ngplosjournals.pagemajik.ai/artanalysis) to test your figure files. NAAS can convert your figure files to the TIFF file type and meet basic requirements (such as print size, resolution), or provide you with a report on issues that do not meet our requirements and that NAAS cannot fix.

After uploading your figures to PLOS’s NAAS tool - https://ngplosjournals.pagemajik.ai/artanalysis, NAAS will process the files provided and display the results in the "Uploaded Files" section of the page as the processing is complete. If the uploaded figures meet our requirements (or NAAS is able to fix the files to meet our requirements), the figure will be marked as "fixed" above. If NAAS is unable to fix the files, a red "failed" label will appear above. When NAAS has confirmed that the figure files meet our requirements, please download the file via the download option, and include these NAAS processed figure files when submitting your revised manuscript.

**Reproducibility:**

To enhance the reproducibility of your results, we recommend that authors of applicable studies deposit laboratory protocols in protocols.io, where a protocol can be assigned its own identifier (DOI) such that it can be cited independently in the future. Additionally, PLOS ONE offers an option to publish peer-reviewed clinical study protocols. Read more information on sharing protocols at https://plos.org/protocols?utm_medium=editorial-email&utm_source=authorletters&utm_campaign=protocols

# Author comment: Author response to Decision Letter 1

**DOI:** 10.1371/journal.pcbi.1014487.r002

15 Apr 2026

Attachment Submitted filename: Reviewer_Comments.pdf

# Peer review: Decision Letter 1

**DOI:** 10.1371/journal.pcbi.1014487.r003

**Authors:** Jeffrey Skolnick, Ferhat Ay, Shaun Mahony

23 Jun 2026

Dear Dr. Gonzalez Camara,

We are pleased to inform you that your manuscript 'Exploring the Structural Lexicon of the Proteome via Metric Geometry' has been provisionally accepted for publication in PLOS Computational Biology.

Before your manuscript can be formally accepted you will need to complete some formatting changes, which you will receive in a follow up email. A member of our team will be in touch with a set of requests.

Please note that your manuscript will not be scheduled for publication until you have made the required changes, so a swift response is appreciated.

IMPORTANT: The editorial review process is now complete. PLOS will only permit corrections to spelling, formatting or significant scientific errors from this point onwards. Requests for major changes, or any which affect the scientific understanding of your work, will cause delays to the publication date of your manuscript.

Should you, your institution's press office or the journal office choose to press release your paper, you will automatically be opted out of early publication. We ask that you notify us now if you or your institution is planning to press release the article. All press must be co-ordinated with PLOS.

Thank you again for supporting Open Access publishing; we are looking forward to publishing your work in PLOS Computational Biology.

Best regards,

Shaun Mahony

Section Editor

PLOS Computational Biology

Ferhat Ay

Section Editor

PLOS Computational Biology

***********************************************************

Reviewer's Responses to Questions

**Comments to the Authors:**

**Please note here if the review is uploaded as an attachment.**

Reviewer #2: My comments have been addressed pretty well. I think the paper is suitable for publication.

Reviewer #3: The authors have satisfactorily responded to my queries and comments.

**********

**Have the authors made all data and (if applicable) computational code underlying the findings in their manuscript fully available?**

The PLOS Data policy requires authors to make all data and code underlying the findings described in their manuscript fully available without restriction, with rare exception (please refer to the Data Availability Statement in the manuscript PDF file). The data and code should be provided as part of the manuscript or its supporting information, or deposited to a public repository. For example, in addition to summary statistics, the data points behind means, medians and variance measures should be available. If there are restrictions on publicly sharing data or code —e.g. participant privacy or use of data from a third party—those must be specified.

Reviewer #2: Yes

Reviewer #3: Yes

**********

PLOS authors have the option to publish the peer review history of their article (what does this mean?). If published, this will include your full peer review and any attached files.

If you choose “no”, your identity will remain anonymous but your review may still be made public.

**Do you want your identity to be public for this peer review?**  For information about this choice, including consent withdrawal, please see our Privacy Policy.

Reviewer #2: No

Reviewer #3: No

# Editor report: Acceptance letter

**DOI:** 10.1371/journal.pcbi.1014487.r004

**Authors:** Jeffrey Skolnick, Ferhat Ay, Shaun Mahony

PCOMPBIOL-D-25-02291R1

Exploring the Structural Lexicon of the Proteome via Metric Geometry

Dear Dr Gonzalez Camara,

I am pleased to inform you that your manuscript has been formally accepted for publication in PLOS Computational Biology. Your manuscript is now with our production department and you will be notified of the publication date in due course.

The corresponding author will soon be receiving a typeset proof for review, to ensure errors have not been introduced during production. Please review the PDF proof of your manuscript carefully, as this is the last chance to correct any errors. Please note that major changes, or those which affect the scientific understanding of the work, will likely cause delays to the publication date of your manuscript.

Soon after your final files are uploaded, unless you have opted out, the early version of your manuscript will be published online. The date of the early version will be your article's publication date. The final article will be published to the same URL, and all versions of the paper will be accessible to readers.

For Research, Software, and Methods articles, you will receive an invoice from PLOS for your publication fee after your manuscript has reached the completed accept phase. If you receive an email requesting payment before acceptance or for any other service, this may be a phishing scheme. Learn how to identify phishing emails and protect your accounts at https://explore.plos.org/phishing.

Thank you again for supporting PLOS Computational Biology and open-access publishing. We are looking forward to publishing your work!

With kind regards,

Janani Seenivasan

PLOS Computational Biology | Carlyle House, Carlyle Road, Cambridge CB4 3DN | United Kingdom ploscompbiol@plos.org | Phone +44 (0) 1223-442824 | ploscompbiol.org | @PLOSCompBiol
